# Ring-Opening Polymerization of Cyclohexene Oxide and Cycloaddition with CO_2_ Catalyzed by Amine Triphenolate Iron(III) Complexes

**DOI:** 10.3390/molecules29092139

**Published:** 2024-05-04

**Authors:** Peng Li, Sixuan Li, Xin Dai, Shifeng Gao, Zhaozheng Song, Qingzhe Jiang

**Affiliations:** 1 State Key Laboratory of Heavy Oil Processing, College of Science, China University of Petroleum, Beijing 102249, China; liyongpeng000@126.com (P.L.); 18841658103@163.com (S.L.); daixin_312@163.com (X.D.); 2 CNPC Engineering Technology R&D Company Ltd., Beijing 102206, China; gaosfdr@cnpc.com.cn; 3 School of International Trade and Economics, University of International Business and Economics, Beijing 100029, China

**Keywords:** cyclohexene oxide, ring-opening polymerization, carbon dioxide, cycloaddition, iron complexes

## Abstract

A series of novel amine triphenolate iron complexes were synthesized and characterized using UV, IR, elemental analysis, and high-resolution mass spectrometry. These complexes were applied to the ring-opening polymerization (ROP) of cyclohexene oxide (CHO), demonstrating excellent activity (TOF > 11050 h^−1^) in the absence of a co-catalyst. In addition, complex C1 maintained the dimer in the presence of the reaction substrate CHO, catalyzing the ring-opening polymerization of CHO to PCHO through bimetallic synergy. Furthermore, a two-component system consisting of iron complexes and TBAB displayed the ability to catalyze the reaction of CHO with CO_2_, resulting in the formation of cis-cyclic carbonate with high selectivity. Complex C4 exhibited the highest catalytic activity, achieving 80% conversion of CHO at a CHO/C4/TBAB molar ratio of 2000/1/8 and a CO_2_ pressure of 3 MPa for 16 h at 100 °C, while maintaining >99% selectivity of cis-cyclic carbonates, which demonstrated good conversion and selectivity.

## 1. Introduction

CO_2_ is a significant greenhouse gas and a renewable C1 feedstock that is abundant, non-toxic, and inexpensive [1,2]. The reaction between CO_2_ and epoxides to produce cyclic carbonates offers a viable technological solution. This reaction not only immobilizes CO_2_ but also yields a high-value-added product. With a 100% atom economy, it is considered a promising green reaction [3,4]. Cyclic carbonates possess desirable properties such as non-toxicity, flame retardancy, high dipole moment, and a high boiling point. They can be utilized as effective clean polar solvents, battery electrolytes [5], antifreeze additives [6], and reaction intermediates [7,8].

Cyclohexene oxide (CHO) is a significant chemical intermediate that can react with CO_2_ to produce cyclic carbonates [9,10,11] and polycarbonates [12,13,14]. It can also form poly(cyclohexene oxide) (PCHO) through ring-opening polymerization (ROP), which results in a rigid aliphatic ring structure in the main chain. PCHO finds wide application in coatings, printing, and sealants. Currently, the ROP of epoxides is primarily conducted through anionic, cationic, and coordination polymerization methods [15]. However, the use of metal complexes as catalysts offers milder reaction conditions and higher catalytic activity, facilitating the production of high molecular weight polyether. Activation of the epoxide and CO_2_ insertion are crucial steps in the reaction between CO_2_ and epoxide, and catalysts that efficiently activate the reaction substrate are vital in this process [16]. Metal complexes can exhibit excellent catalytic properties by modulating the central metal ion and organic ligands. Previous studies have reported the catalytic activity of various metal complexes such as Al [17,18,19,20,21], Zn [22,23], Mn [24], Co [25,26], Fe [15], and Cr [27] in the ROP of epoxides. For instance, Robert et al. utilized porphyrin Mn as a catalyst and obtained trace polymers after a prolonged reaction at 110 °C with a molar ratio of CHO to the catalyst of 1000/1 [28]. Thiam et al. prepared Al complexes that could catalyze the ring-opening polymerization of ethylene oxide (EO), but the monomer conversion was low [29]. Additionally, Coates et al. developed a series of bimetallic cobalt complexes that exhibited good stereoselectivity and catalytic activity for the ring-opening polymerization reactions of various epoxides [30,31,32].

Catalytic systems based on heavy metals like Co, Cr, Ni, and Mn often suffer from heavy metal residues, which significantly limits their application. On the other hand, catalysts containing Al and Zn as central metals are sensitive to moisture and air, requiring strict preparation conditions. In contrast, Fe is an important catalytic material that offers several advantages such as low toxicity, a reasonable price, abundant availability, and sustainability compared to many transition metals [33]. Additionally, the preparation of iron(III) complexes is simple and stable. Moreover, Fe is extensively used as a biocatalyst in nature and plays a crucial role in organic synthesis [34,35,36]. Erkan et al. successfully utilized mixed-valent iron trifluoroacetate complexes to achieve effective ROP of epoxides, although high loading (1 mol%) and the presence of 4 Å molecular sieves were necessary for optimal catalytic performance [15]. Dilworth et al. reported on iron(III) complexes with asymmetric N-capped tripodal NO_3_ ligands, and the catalytic activity of these complexes towards epoxidation of styrene was examined [37]. Kleij et al. synthesized amino triphenolate iron(III) complexes which were able to copolymerize and cycloaddition epoxides with CO_2_ effectively [12,38]. Subsequently, they use amino triphenolate iron(III) complexes for the synthesis of various polyesters [39]. In our previous work, we bridged two amino triphenolate iron(III) moieties through a rigid phenylene linker and studied their use in the alternating copolymerization of CHO/CO_2_ and CHO/PA [40]. Yao et al. used dinuclear aluminum poly(phenolate) complexes as catalysts for cycloaddition of CO_2_ and epoxides [11]. Herein, we present a series of new asymmetric amino triphenolate iron(III) complexes, which exhibit efficient catalytic activity in the ROP of CHO to PCHO without the need for a co-catalyst. Furthermore, the iron complex, in combination with tetrabutylammonium bromide (TBAB), can co-catalyze the cycloaddition reaction of CHO with CO_2_, showcasing different mechanisms of action for the iron-based complexes in different reactions.

## 2. Results and Discussion

### 2.1. Synthesis and Characterization of Fe(III) Amine Triphenolate Complexes

**Scheme 1:**
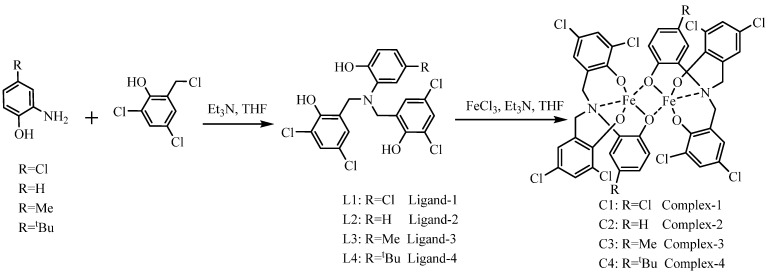
Synthesis roadmap of ligands and complexes.

The ligands L1−L4, featuring diverse substituents, were synthesized via the methods outlined in Scheme 1. Differently substituted 2-hydroxy-5-R-aniline (R=H, Me, ^t^Bu, Cl) was alkylated with 2,4-di-chloro-6-(chloromethyl) phenol, affording the ligands in good yields. Treatment of ligands L1−L4 with a THF solution of iron(III) chloride (one equivalent) in the presence of triethylamine (three equivalents) at 70 °C yielded the corresponding complexes C1–C4. After workup, these iron(III) complexes were isolated by recrystallization as black solids, which are air- and moisture-stable. FT-ICR mass spectrometry, elemental analysis, and UV–vis spectroscopy were carried out to confirm complexes C1−C4 (see ESI, Figures S1–S14).

**Figure 1:**
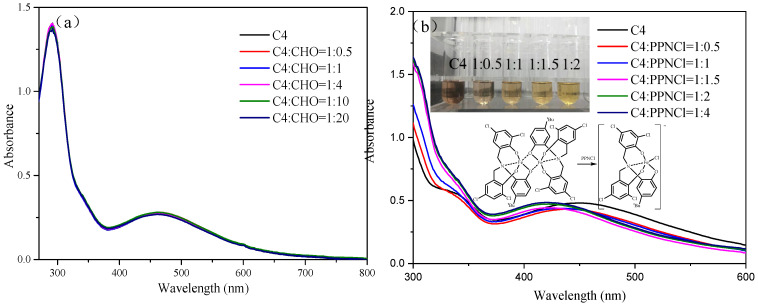
UV spectrum of complex C4 titrated with different equivalent CHO (**a**) and PPNCl (**b**).

The study conducted by Kleij et al. focused on the stability of symmetrical amine triphenolate complexes in the presence of different ligand substituents [38,41]. The researchers observed that the dimerization of the complex was hindered by the presence of tertiary butyl and phenyl groups with large steric hindrance, while the presence of methyl and hydrogen substituents facilitated the dimerization. To confirm the stability of complexes C1–C4, titration experiments were performed using CHO and bis(triphenylphosphine)iminium chloride (PPNCl). The experimental results were obtained by adding excess CHO and PPNCl to iron complexes C1–C4 with different substituents. The UV spectra of C1–C4 did not show significant changes when exposed to excess CHO (Figure S15), and the UV spectrum of complex C4 remained unchanged when titrated with different equivalents of CHO (Figure 1a). However, when C4 was mixed with different equivalents of PPNCl, a decrease in absorption peak near 460 nm and an increase in acromion near 340 nm were observed (Figure 1b). This suggests that all complexes are in a dimeric state. High-resolution mass spectrometry was used to characterize C1 and C4 after mixing with two equivalents of PPNCl, revealing ion peaks with *m*/*z* values of 580.8169 and 602.9186 in the negative ion mode, which were attributed to [C_20_H_11_Cl_5_FeNO_3_+Cl]^−^ and [C_24_H_20_Cl_4_FeNO_3_+Cl]^−^, respectively (Figure S16). Further experiments showed that PPNCl can disrupt the dimeric form of the iron complex, causing it to exist in the monomeric form during synergistic catalysis. This is why the catalytic system loses its high activity in the presence of PPNCl. These results provide insights into the behavior of the iron complexes and the effects of PPNCl on their catalytic activity.

### 2.2. Catalytic Performance of Complexes C1–C4 for the Ring-Opening Polymerization of CHO

**Scheme 2:**
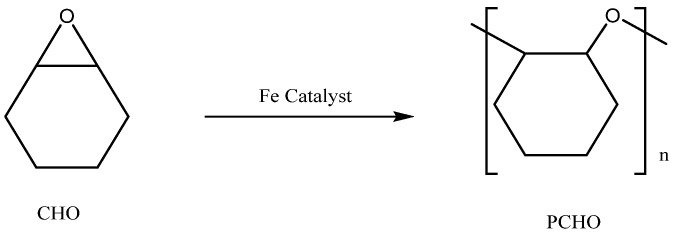
Ring-opening polymerization of CHO catalyzed by iron catalyst.

The catalytic performance of iron complexes in the ROP of CHO to PCHO was investigated (Scheme 2). The reaction time was measured in terms of rotor standstill time, with increasing system viscosity due to PCHO formation. This study found that the C1–C4 complexes exhibited excellent catalytic performance at reaction temperatures of 25 °C and 35 °C. The TOF values demonstrated a ranking of C1 > C2 > C4 > C3. Specifically, complex C1 with electron-withdrawing substituents (R=Cl) displayed the most superior catalytic performance, indicating the favorable impact of these substituents on catalytic reactions. Conversely, electron donating substituents (R=Me or ^t^Bu) were associated with lower catalytic activity, resulting in a reduction in the number-average molecular weight of the polymer and an increase in the dispersion coefficient. While increasing temperature accelerates the reaction rate, it does not improve polymer yield due to a rapid local viscosity increase that impedes the continuation of the reaction [18]. Entry 9 showed high sensitivity of the ring-opening polymerization of CHO to air or moisture, while entry 10 indicated that PPNCl cannot catalyze the ring-opening of CHO when added to the system, distinguishing it from the work of Chatterjee et al., who used porphyrin Cr to catalyze the ROP of PO [42]. Overall, the results suggest that C1 can catalyze the ROP of CHO without requiring the synergistic action of other nucleophilic reagents.

This study aimed to investigate the impact of catalyst concentration on the TOF at various molar ratios of CHO/C1 and temperatures (Table 1). At 25 °C, increasing the molar ratio from 5000/1 to 10,000/1 resulted in a 44.5% decrease in TOF, from 22,773 h^−1^ to 12,623 h^−1^. Further increasing the molar ratio to 20,000/1 led to a 48% decrease in TOF, from 12,623 h^−1^ to 6062 h^−1^. On the other hand, at 35 °C, the TOF value only decreased by 13.9%, from 19,950 h^−1^ to 17,165 h^−1^, when the molar ratio was increased from 10,000/1 to 20,000/1. However, increasing the molar ratio to 40,000/1 resulted in a significant 66% decrease in TOF, from 17,165 h^−1^ to 5780 h^−1^. These findings suggest that the reaction system can maintain high catalytic activity at a certain ratio despite concentration effects, and raising the temperature accelerates molecular movement, enabling the catalytic system to maintain high activity at low loading.

In terms of polymerization, the polymers had an average molecular weight ranging from 19.1 to 25.3 kg/mol, with a dispersity between 1.7 and 1.8, which was lower than the theoretical average molecular weight. This can be attributed to the rapid growth of polymer chains within a short period, resulting in increased local viscosity of the polymerization system and hindering chain growth. Increasing the temperature enhances the chain transfer rate, thereby reducing the average molecular weight of the polymer and increasing the dispersity.

The influence of temperature on polymerization activity was particularly significant in the early stages of the reaction. When the temperature increased from 25 °C to 45 °C, the catalytic activity significantly increased, with the TOF value rising from 12,623 h^−1^ to 38,000 h^−1^. Further increasing the temperature to 55 °C resulted in a TOF value of 42,911 h^−1^. However, as the reaction temperature increased, the average molecular weight of the polymer decreased. The number-average molecular weight of the polymer is primarily determined by the ratio of the chain growth rate constant to the chain transfer rate constant [43]. Typically, the activation energy of chain transfer is higher than that of chain growth in polymerization reactions. Therefore, increasing the temperature accelerates the chain transfer rate, leading to a reduction in the molecular weight of the polymer and an increase in the dispersion coefficient.

To investigate the impact of solvents on polymerization reactions (Table 2), various solvents were examined to enhance the reaction yield and reduce viscosity. The findings indicated that, with the exception of THF, the selected solvents increased the reaction yield to some extent while decreasing the TOF. THF, an oxygen heterocyclic compound, can coordinate with the metal center of iron and compete with CHO, hindering coordination between CHO and the metal center, thereby preventing polymerization reactions. When dichloromethane was used as the reaction solvent with a fixed volume of 1 mL, the yield of the reaction system initially increased with extended time and then stabilized. This suggests that appropriate solvent selection can reduce the viscosity of the reaction system and improve the yield. However, as polymerization time increased, the polymerization activity decreased primarily due to the increased viscosity of the reaction system and the decrease in the number of monomers, resulting in reduced activity. Under the same conditions, polymerization reactions using dichloromethane and 1,2-dichloromethane solvents exhibited higher activity compared to toluene and hexane solvents. Toluene, being an electrically neutral solvent, weakened the activity of the active center, reducing catalytic activity and hindering polymer growth chain formation, ultimately leading to a lower molecular weight. Consequently, the number-average molecular weight of the polymerization products with toluene as the solvent (21.7 kg/mol) was lower than that with dichloromethane as the solvent (25.5 kg/mol) (entry 3 vs. entry 5). 

### 2.3. Reaction Kinetic Studies

**Figure 2:**
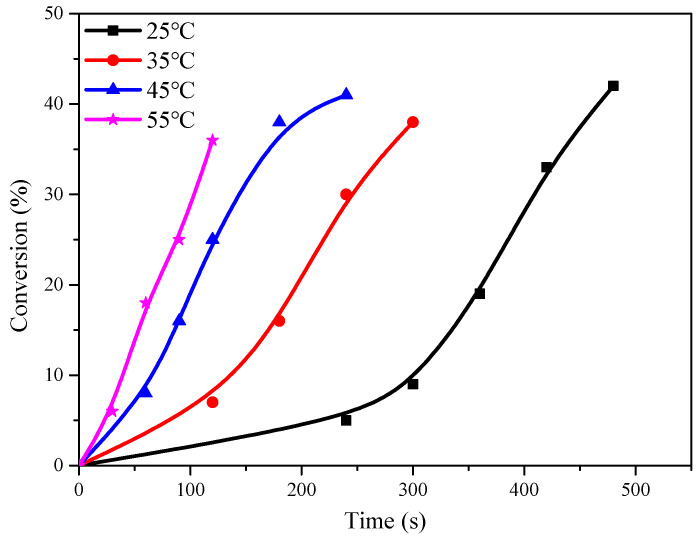
Relationship between CHO conversion and time at different temperatures.

The polymerization experiments were conducted using C1 as a catalyst, with a CHO/Cat molar ratio of 10,000/1, under identical conditions of temperature and time. The results showed that the temperature had a significant effect on the conversion rate of CHO during the initial phase of the reaction, but this effect diminished as the process progressed (Figure 2). After 5 min of reaction at 25 °C, the conversion of CHO was less than 10%. However, by extending the reaction time to 8 min, the conversion could reach up to 40%. As the reaction proceeded, the released heat raised the temperature of the reaction system, overcoming the influence of the ambient temperature. Although high yields were obtained at different temperatures with short reaction times, the concentration of CHO continuously decreased, and the viscosity of the system increased due to the generation of polymerization products. These variations resulted in the reduction in the active center and the growth rate of yield, as they hindered the migration of the reaction monomer and catalyst.

**Figure 3:**
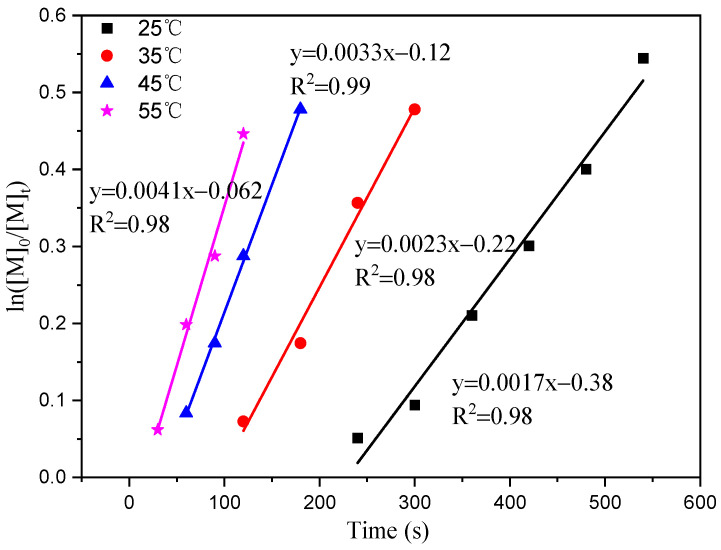
First-order ln([M]_0_/[M]_t_) vs. time plot at different temperatures.

**Figure 4:**
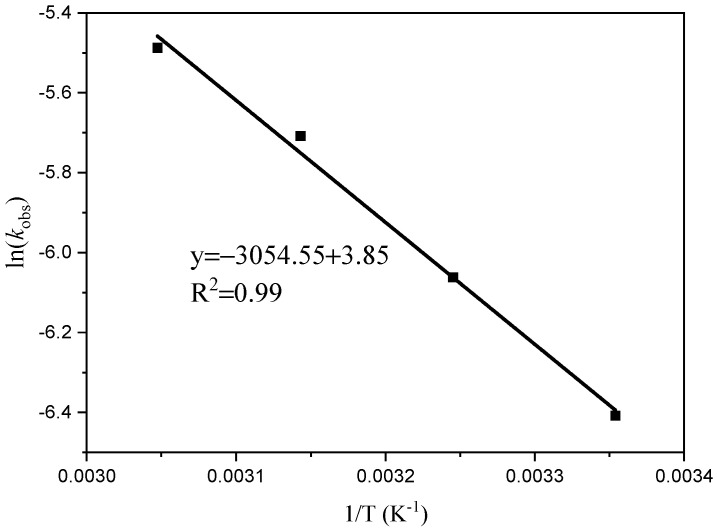
Arlenius plot for the formation of PCHO.

The results of this study indicate that ln[M]_0_/[M]_t_ is linear over time (t). Figure 3 illustrates that polymerization shows a first-order response to monomer concentration at a specific time. The apparent rate constants (k) were determined at temperatures of 25 °C, 35 °C, 45 °C, and 55 °C, resulting in values of 0.0017, 0.0023, 0.0033, and 0.0041 h^−1^, respectively. Plotting ln (k) against 1/T at different reaction temperatures yielded a slope of −3054.55 (Figure 4). By using the Arrhenius formula, the apparent activation energy of the polymerization reaction was calculated to be 25.40 kJ/mol. In comparison, Ambrose et al. utilized Schiff Cr to catalyze the ROP of CHO with an activation energy of 64.0 kJ/mol [27], and the activation energy of the reaction catalyzed by C1 under the same conditions was found to be lower. 

### 2.4. Catalytic Mechanism

**Scheme 3:**
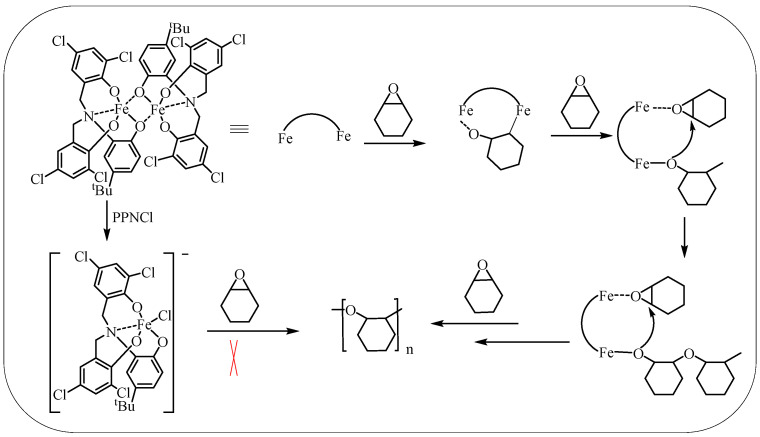
Proposed mechanism for CHO ring-opening polymerization mediated by iron(III) complex.

The proposed mechanism for CHO ring-opening polymerization catalyzed by dinuclear iron complexes is discussed in this study (Scheme 3). Previous studies have also reported similar mechanism [16]. It involves the coordination and activation of CHO molecules with an iron center, followed by the promotion of ring-opening of the activated CHO by another adjacent iron metal center. The resulting alkoxy group then triggers the ring-opening of CHO bound to the adjacent iron center, leading to an increase in the polymer chain. It is worth noting that when the concentration of the iron complex is high, adjacent iron metal centers may originate from the same catalyst or adjacent catalysts. This is a significant factor contributing to the decrease in catalytic activity when the solvent is added or the catalyst concentration is reduced, as the intermolecular bimetallic synergism is weakened. However, even at reduced catalyst concentrations (1/20,000), the TOF can reach up to 6062 h^−1^ due to the intramolecular bimetallic mechanism.

### 2.5. Analysis of Polymerization Products

The presence of a characteristic absorption peak at 1089 cm^−1^ in the infrared spectrum suggests the formation of a polyether bond structure (Figure S17). Further insights into the structure were obtained from the NMR hydrogen spectrum of PCHO. The spectrum revealed distinct chemical shifts for the H protons on the last methyl group (δ 3.3–3.5, m, 2H) and the submethyl group (δ 3.37, δ 3.43, and δ 3.53) (Figure S18). These chemical shifts correspond to the polymerization products of homogeneity, isogeneity, and random structure [44]. Notably, the NMR spectra of the polymerized products showed three different structures, indicating that the iron complex used for the ring-opening polymerization of CHO lacked selectivity. This finding is consistent with the results of Sokolovicz et al. [45], who observed a similar lack of selectivity in the reaction catalyzed by Zn-based complexes to produce PCHO.

The Tg of PCHO with varying molecular weights ranges from 63.7 to 67.4 °C (Figure S19), indicating that molecular weight has minimal effect on Tg. This is in line with the previous literature reporting Tg values of polyethers with different molecular weights [18,46]. Thermal stability analysis was performed on PCHO, which revealed that decomposition began at approximately 350 °C and was complete at around 450 °C (Figure S20). According to Zhao et al. the weight loss temperature range of PCHO catalyzed by palladium chloride under thermal initiation is 320–400 °C [47] due to its average molecular weight of 4.5 kg/mol and dispersity of 2.20. The PCHO catalyzed by iron complexes has a higher molecular weight and lower dispersity, indicating that PCHO generated by coordination polymerization with high molecular weight and low dispersion possesses greater thermodynamic stability, a wider processing temperature range, and broader application potential. In addition, MALDI-TOF was attempted to analyze the end groups of the polymer product PCHO, but unfortunately, ideal results could not be obtained.

### 2.6. Catalytic Performance of Complexes C1–C4 for the Reaction of CHO with CO_2_

**Scheme 4:**
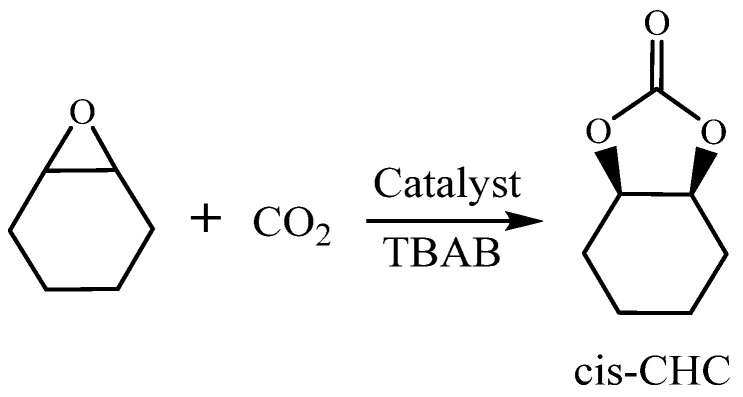
Cycloaddition reaction of CHO/CO_2_.

Carmine Capacchione et al. [36] reported that bis-thioether-phenolate Fe(II) and Fe(III) complexes catalyze cycloaddition reactions of CO_2_ with different epoxides. Furthermore, the performance of the catalytic reaction between CHO and CO_2_ using complexes C1–C4 was investigated (Scheme 4). We examined the catalytic effect of different co-catalysts, specifically PPNCl (entry 3) and TBAB (entries 7–9) in Table 3. Three different molar ratios of iron complex C4 and TBAB (1/2, 1/4, and 1/8) were tested. When TBAB was used as a co-catalyst, it showed a better catalytic effect compared to PPNCl. This is because TBAB has a stronger nucleophilicity and departure ability due to the presence of Br^−^ compared to Cl^−^ [48]. Additionally, PPNCl has greater site resistance compared to TBAB. It is worth noting that the conversion rate was only 17% when TBAB was used alone (entry 2) but increased to 71–80% with the addition of iron complexes while maintaining a remarkable selectivity for cis-cyclic carbonate. The conversion of CHO increased as the level of co-catalyst TBAB increased under the same conditions. Furthermore, the selectivity of PCHO and cyclic esters increased when the molar ratio was changed from 1/2 to 1/4 (entry 5 vs. entry 6), resulting in 2% polyether content at a C4/TBAB molar ratio of 1/2. A 99% selectivity of cyclic carbonate was achieved at different molar ratios of C4/TBAB. The presence of Cl^−^ and Br^−^ in the reaction system hindered the attack of growing polymer chains on the CHO molecules, leading to an intramolecular ring closure reaction via the SN_2_ mechanism and generating cis-cyclic carbonates. This explains the increased selectivity of cyclic esters with the co-catalyst [49].

The performance differences of the C1–C4 catalysts in CHO polymerization with CO_2_ were examined under identical conditions. Table 3 shows that all four iron complexes, despite having different substituents, demonstrated exceptional catalytic effects and selectivity. The catalytic activities of the four catalysts followed the order C4 > C3 > C2 > C1 which is opposite to the ROP of CHO. This indicates significant variations between the CHO/CO_2_ reaction process and CHO due to the simultaneous occurrence of CHO molecule ring-opening and CO_2_ insertion in the former. The ROP of CHO is mainly influenced by the ease of CHO molecule ring-opening, as opposed to the CHO/CO_2_ reaction. Moreover, complex C4 with electron-donating substituents achieved the highest CHO conversion, while complex C1 with electron-withdrawing groups displayed the lowest catalytic activity (entry 7 vs. entry 8). These results suggest that an excessively high Lewis acidity does not contribute to enhanced catalytic activity.

This study examined the impact of reaction conditions on CHO conversion. The results presented in Table 4 show that increasing the reaction time led to a gradual increase in conversion. After 4 h, the conversion reached 37%, and after 16 h, it reached 80%. It is worth noting that the reaction rate remained constant throughout this time period, despite a decrease in monomer concentration (entries 2–5). Furthermore, this study investigated the effect of temperature on conversion. The findings revealed that temperature had a more significant influence on conversion. At 100 °C, the conversion rate was 65%, but it decreased to 47% at 80 °C, resulting in 2% polycarbonate production. However, when the temperature was raised to 120 °C, the conversion reached 85% within 12 h. This phenomenon can be attributed to the thermodynamic nature of cyclic carbonate, where increased temperature enhances both conversion and selectivity [50].

The effect of CO_2_ pressure on the cycloaddition reaction was investigated. Similar to previous studies on epoxide/CO_2_ coupling reactions [51,52], the reaction yield and TOF values initially increased with increasing pressure, but gradually decreased. This trend can be attributed to the formation of amphiphilic complexes when TBAB reacts with CO_2_ at high pressure, leading to a reduction in catalytic activity [53]. The impact of pressure on conversion was relatively minor compared to temperature. A pressure of 0.1 MPa resulted in 30% conversion over 12 h. Notably, the presence of 3% polyether in the resulting polymer decreased when the pressure increased to 1.5 MPa, indicating that high-pressure environments discourage polyether formation. Entries 3 and 9–11 demonstrated that CHO conversion increased with increasing CO_2_ pressure within the range of 0–4.5 MPa. However, further increasing the pressure beyond 3 MPa only provided a marginal increase in conversion (65% vs. 68%). This is because, at high pressures, the rate-limiting step in the reaction is no longer the introduction of CO_2_. Excessive pressure can lead to an influx of CO_2_ molecules into the liquid phase, resulting in a decrease in the reaction rate [54].

### 2.7. Kinetic Studies of Reactions

**Figure 5:**
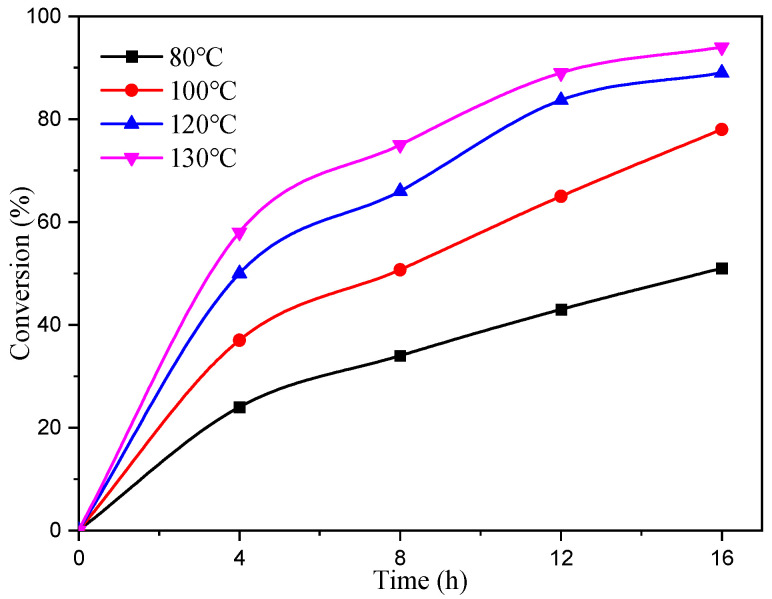
Relationship between CHO conversion and time at different temperatures.

The experiment utilized complex C4 as a catalyst, maintaining CHO/Cat/TBAB molar ratios at a constant value of 2000/1/8. The reaction was conducted at different temperatures and durations, and the resulting conversion of CHO over time is visually depicted in Figure 5. The obtained data clearly demonstrated the significant impact of temperature on CHO conversion. After 4 h of reaction, the conversion rates of CHO at 80 °C, 100 °C, 120 °C, and 130 °C were 23%, 36%, 45%, and 58%, respectively. Moreover, the conversion rate at 120 °C was twice as high as that observed at 80 °C for the same reaction time. Initially, the reaction exhibited faster rates, which gradually slowed down due to the formation of liquid cyclic carbonate. Consequently, the ratio of CHO to catalyst decreased, leading to a decline in the reaction rate.

**Figure 6:**
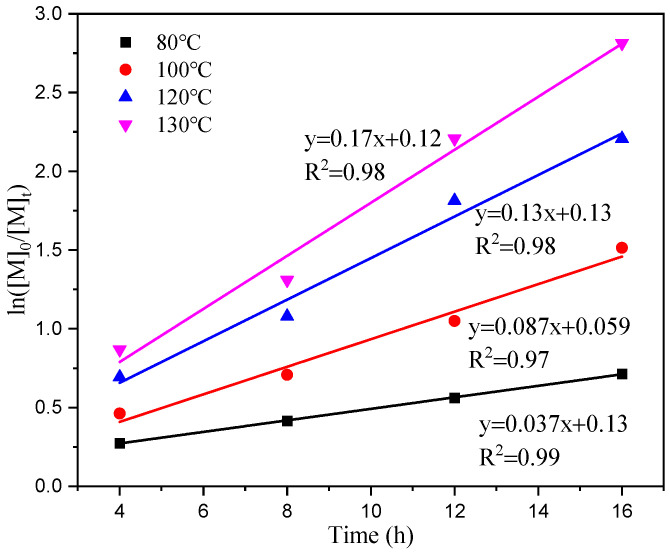
First-order ln([M]_0_/[M]_t_) vs. time plot at different temperatures.

**Figure 7:**
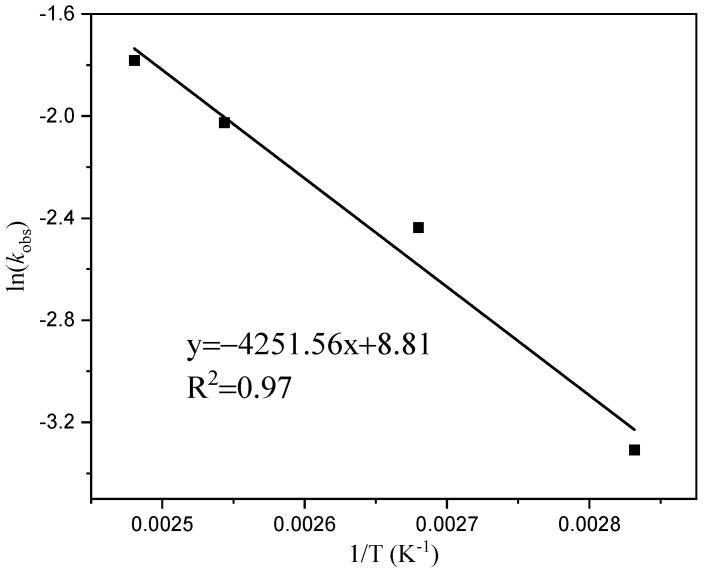
Arlenius plot for the formation of cyclic esters.

Figure 6 illustrates a linear relationship between time (t) and the table ln[M]_0_/[M]_t_, indicating that the primary reaction rate constants (k) at temperatures of 80 °C, 100 °C, 120 °C, and 130 °C are 0.037, 0.087, 0.13, and 0.17 h^−1^, respectively, based on a given monomer concentration and time. The rate of dissociation increases with an increasing temperature reaction, and it should decrease the induction period of the reaction. In Figure 7, when plotting the reaction rate constants (k) at different temperatures against the horizontal coordinate of temperature (1/T), we observe a linear correlation between ln (k) and 1/T. The slope of this line is −4251.56. By utilizing the Arrhenius formula, an apparent activation energy of 35.35 kJ/mol for the reaction can be determined.

### 2.8. Mechanism of the C4/TBAB-Catalyzed CHO/CO_2_ Cycloaddition Reaction

**Scheme 5:**
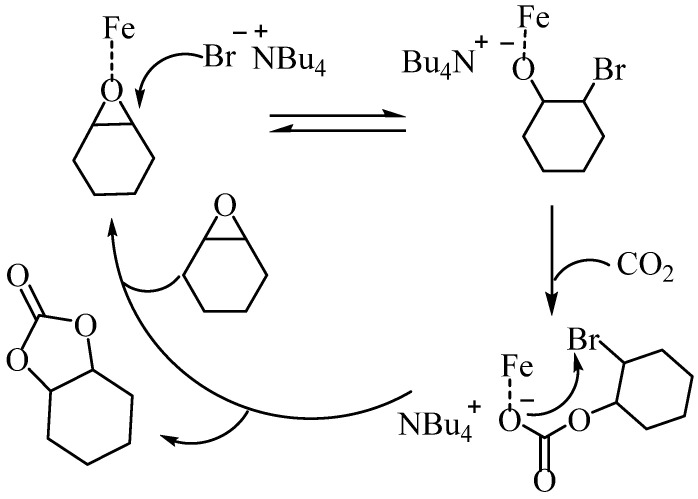
Proposed mechanism of cyclic carbonate synthesis.

To confirm the status of the iron-based complexes in the two-component catalytic system consisting of iron-based complexes and TBAB, the changes in the UV spectra of C4 were analyzed using UV titration (Figure S21). The analysis revealed that upon the addition of TBAB, the absorption peak near 460 nm continuously decreased and shifted towards the blue, while the shoulder peak near 340 nm continuously increased. However, there was negligible change in the spectrum when the equivalent of TBAB was increased from 1.5 to 2, indicating that the system stabilized at this equivalence. Furthermore, when the complex C4 was mixed with two equivalents of TBAB and characterized via mass spectrometry, an ion peak with *m*/*z* of 646.8676 corresponding to [C_24_H_20_Cl_4_FeNO_3_+Br]^−^ was detected (Figure S22). These results suggest that the co-catalyst TBAB can disrupt the dimeric form of the iron complex. Consequently, in the catalytic process, binuclear complexes first form mononuclear active substances with the co-catalyst before further catalyzing the reaction. The potential reaction mechanism is presented in Scheme 5 [11]. Initially, CHO is activated upon coordination with the central metal of the iron complex. Subsequently, CHO opens the ring upon nucleophilic attack by bromide ions, followed by CO_2_ insertion into the Fe-O bond to create the carbonate. Finally, intramolecular cyclization leads to the formation of cyclic carbonate.

## 3. Experimental Section

### 3.1. General Experimental Conditions

All polymerization and cycloaddition reactions were carried out under an atmosphere of dry argon using the standard Schlenk technique. Unless stated otherwise, all solvents and reagents were purchased from commercial sources and were used as received. Cyclohexene oxide (98%) was purchased from Aladdin, dried over CaH_2_ for 36 h and distilled fresh under an argon atmosphere prior to use. Toluene was distilled from sodium/benzophenone under argon. Research grade CO_2_ was purchased from Yong Sheng Gases Technology Co. Beijing (Beijing, China) (99.995%) and was used as received. 2,4-dichloro-6-(chloromethyl)phenol was synthesized following procedures described in the literature [55]. Deuterated chloroform (+99.7 atom%) as solvent for the samples measured by ^1^H and ^13^C NMR was purchased from Cambridge Isotope Laboratories. NMR spectra were recorded on a Bruker Avance III 500 MHz spectrometer at ambient temperature. ^1^H NMR chemical shifts were referenced in ppm from the peak of internal tetramethylsilane; ^13^C NMR chemical shifts are referenced to the standard shifts of the corresponding deuterated reagents. Mass spectrometry was taken with a Thermo Scientific Orbitrap mass spectrometer (Thermo Scientific, Waltham, MA, USA). UV–vis spectra were recorded on a Shimadzu UV-2600 spectrophotometer. FT-IR measurements were obtained on a Thermo Scientific Nicolet iS50 FT-IR spectrometer (Thermo Scientific, Waltham, MA, USA). Elemental analyses of iron(III) complexes were performed by using an Elementar vario EL cube instrument. The molecular weight and dispersity distribution of polymer samples were determined by gel permeation chromatography on a Prominence LC-20 AD liquid chromatograph coupled with a RID-20 detector system and THF as the eluent at a flow rate of 1 mL/min at 35 °C. Narrow *M*n polystyrene standards were used for calibration. Glass transition temperatures (Tg) were measured using a Netzsch DSC214 (Netzsch, Selb, Germany). Samples were weighed into 40 μL aluminum pans and subjected to two heating cycles from 35 °C to 180 °C at a rate of 10 °C/min. Tg of polymers were determined from the second heating.

### 3.2. Synthesis of Ligands and Characterization

The four ligands 1–4 were synthesized via a general procedure: Under argon atmosphere, a solution of 2-hydroxyl-5-R-aniline (R=Cl, H, Me, ^t^Bu) in THF was added to a solution of 2,4-dichloro-6-chloromethylphenol in THF, and then triethylamine was added to the reaction flask. After heating at reflux for 10 h, the white precipitate was removed by filtration after cooling. The solvent was removed with a rotary evaporator to obtain the crude product and was purified by column chromatography to obtain the target ligand. 2-hydroxy-5-R-aniline:2,4-dichloro-6-chloromethylphenol:trimethylamine in the stoichiometric ratio of 1:2:2.

#### 3.2.1. Synthesis of Ligand-1 (R=Cl)

According to the general procedure, a solution of 2-hydroxy-5-chloro-aniline (0.72 g, 5 mmol) in THF (20 mL) was added dropwise to a stirred solution of 2,4-dichloro-6-(chloromethyl)phenol (2.56 g, 10.0 mmol) in THF (20 mL) followed by triethylamine (1.4 mL, 10.07 mmol) under argon atmosphere. A white solid had formed, and the reaction mixture was refluxed for 10 h. The crude product was purified by column chromatography (silica, eluent: petroleum ether/ethyl acetate: 2/1) to yield Ligand-1 (1.90 g, 77%). ^1^H NMR (500 MHz, CDCl_3_) δ 7.22 (d, *J* = 2.3 Hz, 2H, ArH), 7.15 (d, *J* = 2.2 Hz, 1H, ArH), 7.02 (dd, *J* = 10.6, 2.2 Hz, 3H, ArH), 6.86 (d, *J* = 8.6 Hz, 1H, ArH), 4.09 (d, *J* = 12.4 Hz, 4H, NCH_2_Ar). ^13^C NMR (126 MHz, CDCl_3_) δ 150.71, 149.15, 136.58, 129.21, 128.69, 127.00, 125.16, 125.00, 124.75, 122.66, 121.14, 117.13, 55.27. FT-IR (KBr, cm^−1^): 3245 (s), 2860 (v), 1602 (w), 1578 (vw), 1501 (s), 1473 (vs), 1418 (m), 1345 (m), 1285 (m), 1214 (s), 1181 (w), 1127 (w), 1097 (vw), 980 (w), 948 (w), 926 (vw), 862 (s), 814 (m), 781 (w), 730 (s), 664 (w), 649 (m), 568 (vw), 518 (w), 464 (w). UV–vis (CH_2_Cl_2_) λ max in nm: 229 nm, 289 nm.

#### 3.2.2. Synthesis of Ligand-2 (R=H)

Yield: 70%. ^1^H NMR (500 MHz, CDCl_3_) δ 7.21 (d, *J* = 2.5 Hz, 2H, ArH), 7.15 (dd, *J* = 7.9, 1.3 Hz, 1H, ArH), 7.05 (td, *J* = 8.0, 1.4 Hz, 1H, ArH), 7.00 (d, *J* = 2.5 Hz, 2H, ArH), 6.92 (dd, *J* = 8.1, 1.4 Hz, 1H, ArH), 6.85 (td, *J* = 7.9, 1.4 Hz, 1H, ArH), 4.13 (s, 4H, NCH_2_Ar). ^13^C NMR (126 MHz, CDCl_3_) δ 151.66, 149.54, 135.05, 129.16, 128.70, 127.06, 125.01 (d, *J* = 15.8 Hz), 122.11, 121.24, 120.57, 116.38, 55.54. FT-IR (KBr, cm^−1^): 3509 (m), 3341 (s), 3082 (m), 2918 (w), 1737 (w), 1602 (m), 1584 (w), 1507 (w), 1461 (vs), 1415 (vw), 1379 (m), 1343 (m), 1317 (m), 1262 (m), 1234 (w), 1210 (m), 1167 (s), 1105 (s), 1087 (vw), 1045 (w), 960 (m), 911 (vw), 865 (s), 836 (w), 766 (s), 729 (s), 711 (w), 578 (w), 513 (m), 425 (w). UV–vis (CH_2_Cl_2_) λ max in nm: 234 nm, 283 nm.

#### 3.2.3. Synthesis of Ligand-3 (R=Me)

Yield: 72%. ^1^H NMR (500 MHz, CDCl_3_) δ 7.20 (d, *J* = 2.3 Hz, 2H, ArH), 7.02–6.92 (m, 3H, ArH), 6.83 (dd, *J* = 18.0, 8.1 Hz, 2H, ArH), 4.11 (s, 4H, NCH_2_Ar), 2.25 (s, 3H, CH_3_). ^13^C NMR (126 MHz, CDCl_3_) δ 149.61, 149.13, 134.84, 129.94, 129.06, 128.67, 127.48, 125.12, 124.85, 122.63, 121.25, 116.10, 55.64, 20.81. FT-IR (KBr, cm^−1^): 3374 (vs), 3069 (m), 2851 (m), 1728 (w), 1604 (m), 1577 (w), 1512 (vs), 1467 (vs), 1352 (s), 1286 (s), 1241 (w), 1217 (m), 1172 (m), 1132 (m), 1098 (w), 985 (w), 955 (m), 929 (w), 864 (s), 813 (m), 784 (m), 737 (s), 668 (vw), 594 (w), 571 (w), 526 (w). UV–vis (CH_2_Cl_2_) λ max in nm: 232 nm, 285 nm.

#### 3.2.4. Synthesis of Ligand-4 (R=^t^Bu)

Yield: 82%. ^1^H NMR (500 MHz, CDCl_3_) δ 7.20 (d, *J* = 2.4 Hz, 2H, ArH), 7.10 (d, *J* = 2.2 Hz, 1H, ArH), 7.06–6.99 (m, 3H, ArH), 6.85 (d, *J* = 8.4 Hz, 1H, ArH), 4.16 (s, 4H, NCH_2_Ar), 1.24 (s, 9H, C(CH_3_)_3_). ^13^C NMR (126 MHz, CDCl_3_) δ 149.75, 148.79, 143.37, 133.90, 129.21, 128.70, 125.04, 124.77, 123.44, 121.40, 119.41, 115.70, 55.23, 34.27, 31.42. FT-IR (KBr, cm^−1^): 3343 (s), 3038 (w), 2963 (s), 2862 (w), 1732 (vw), 1606 (m), 1515 (s), 1468 (vs), 1427 (w), 1365 (vw), 1350 (m), 1288 (m), 1214 (s), 1168 (s), 1134 (w), 1084 (w), 983 (w), 950 (m), 927 (w), 863 (s), 778 (w), 730 (s), 664 (w), 567 (w), 456 (vw). UV–vis (CH_2_Cl_2_) λ max in nm: 231 nm, 286 nm.

### 3.3. Synthesis of Complexes and Characterization

The iron(III) complexes **1**–**4** were prepared via a general procedure: Under argon atmosphere, a solution of anhydrous FeCl_3_ in THF was added to a solution of ligands d1–d4 in THF, and then, triethylamine was added to the reaction flask. The resulting mixture was refluxed for 8 h and then filtered through Celite. The solvent was removed with a rotary evaporator to obtain the crude product, and the target complexes were obtained after purification. The molar ratio of ligand:anhydrous FeCl_3_:triethylamine was 1:1:3.

#### 3.3.1. Synthesis of Complex-**1** (R=Cl)

A solution of FeCl_3_ (0.16 g, 1.0 mmol) in THF (15 mL) was added dropwise to a stirred solution of Ligand-1 (0.49 g,1.0 mmol) in THF (15 mL) under an argon atmosphere, followed by triethylamine (0.42 mL, 3.0 mmol). The resulting mixture was refluxed for 8 h and then filtered through Celite. After evaporation of the solvent, the crude product was recrystallized from tetrahydrofuran/acetonitrile to give complex-**1** as a black solid (0.39 g, 70%). HRMS (ESI, *m*/*z*): Monoclear [M+Na]^+^ Calcd for [C_20_H_11_Cl_5_FeNO_3_+Na]^+^: 568.8394, found: 568.8394; Dinuclear [2M+Na]^+^ Calcd for [C_40_H_22_Cl_10_Fe_2_N_2_O_6_+Na]^+^: 1114.6895, found: 1114.6889. FT-IR (KBr, cm^−1^): 2977 (m), 1568 (w), 1553 (w), 1481 (s), 1456 (vs), 1366 (m), 1325 (m), 1283 (s), 1251 (w), 1221 (w), 1181 (s), 1124 (m), 1045 (m), 864 (s), 834 (vw), 823 (w), 768 (m), 752 (m), 702 (m), 650 (w), 618 (w), 599 (m), 564 (w), 530 (m), 455 (m). UV–vis (CH_2_Cl_2_) λ max in nm: 232 nm, 288 nm, 336 nm, 435 nm. Anal. calcd for C_40_H_22_Cl_10_Fe_2_N_2_O_6_: C, 43.96; H, 2.03; N, 2.56. Found: C, 44.66; H, 2.15; N, 2.64.

#### 3.3.2. Synthesis of Complex-**2** (R=H)

Yield: 74%. HRMS (ESI, *m*/*z*): Monoclear [M+Na]^+^ Calcd for [C_20_H_12_Cl_4_FeNO_3_+Na]^+^: 534.8783, found: 534.8784; Dinuclear [2M+Na]^+^ Calcd for [C_40_H_24_Cl_8_Fe_2_N_2_O_6_+Na]^+^: 1046.7675, found: 1046.7710. FT-IR (KBr, cm^−1^): 3228 (m), 2923 (w), 1589 (s), 1555 (w), 1484 (m), 1458 (vs), 1364 (m), 1320 (m), 1283 (s), 1254 (m), 1221 (w), 1179 (m), 1111 (w), 1039 (w), 862 (s), 828 (m), 764 (s), 747 (s), 603 (m), 586 (w), 529 (m), 456 (m). UV–vis (CH_2_Cl_2_) λ max in nm: 232 nm, 281 nm, 328 nm, 424 nm. Anal. calcd for C_40_H_24_Cl_8_Fe_2_N_2_O_6_: C, 46.92; H, 2.36; N, 2.74. Found: C, 47.32; H, 2.42; N, 2.78.

#### 3.3.3. Synthesis of Complex-**3** (R=Me)

Yield: 80%. HRMS (ESI, *m*/*z*): Monoclear [M+Na]^+^ Calcd for [C_21_H_14_Cl_4_FeNO_3_+Na]^+^: 548.8940, found: 548.8941; Dinuclear [2M+Na]^+^ Calcd for [C_42_H_28_Cl_8_Fe_2_N_2_O_6_+Na]^+^: 1074.7988, found: 1074.7993. FT-IR (KBr, cm^−1^): 3420 (m), 2978 (w), 2915 (w), 1653 (m), 1557 (w), 1496 (s), 1457 (vs), 1364 (w), 1320 (m), 1280 (s), 1258 (w), 1218 (m), 1180 (s), 1120 (w), 1044 (w), 929 (vw), 861 (m), 835 (w), 819 (m), 763 (s), 668 (vw), 618 (w), 602 (m), 564 (w), 539 (m), 509 (vw), 455 (s). UV-Vis (CH_2_Cl_2_) λ max in nm: 232 nm, 283 nm, 329 nm, 441 nm. Anal. calcd for C_42_H_28_Cl_8_Fe_2_N_2_O_6_: C, 47.95; H, 2.68; N, 2.66. Found: C, 47.92; H, 2.73; N, 2.66.

#### 3.3.4. Synthesis of Complex-**4** (R=^t^Bu)

Yield: 79%. HRMS (ESI, *m*/*z*) Monoclear [M+Na]^+^ Calcd for [C_21_H_14_Cl_4_FeNO_3_+Na]^+^: 590.9409, found: 590.9413; Dinuclear [2M+Na]^+^ Calcd for [C_48_H_40_Cl_8_Fe_2_N_2_O_6_+Na]^+^: 1158.8927, found: 1158.8942. FT-IR (KBr, cm^−1^): 3425 (m), 2559 (m), 2864 (w), 1584 (m), 1495 (s), 1456 (vs), 1361 (vs), 1437 (vs), 1407 (m), 1387 (w), 1363 (m), 1309 (m), 1295 (w), 1259 (vs), 1231 (vw), 1160 (s), 1071 (s), 1029 (s), 898 (m), 877 (s), 859 (s), 818 (vs), 771 (s), 760 (s), 697 (s), 656 (vw), 624 (s), 604 (vs), 570 (m), 557 (s), 547 (s), 509 (s), 492 (m), 460 (vw), 420 (m), 403 (m). UV–vis (CH_2_Cl_2_) λ max in nm: 234 nm, 284 nm, 333 nm, 448 nm. Anal. calcd for C_48_H_40_Cl_8_Fe_2_N_2_O_6_: C, 50.74; H, 3.55; N, 2.47. Found: C, 50.23; H, 3.63; N, 2.41.

### 3.4. General Procedure for the Ring-Opening Polymerization of Cyclohexene Oxide

An iron complex was added to a pre-dried 10 mL thick-walled glass reaction tube in a specific ratio. Under the protection of argon gas, CHO (2 mL, 19.7 mmol) was then introduced into the reaction mixture using a syringe. The reaction tube was placed in a preheated oil bath and stirred for a predetermined period. After the reaction was completed, a sample was taken from the reaction tube for ^1^H NMR to determine the conversion of CHO. The remaining reaction mixture was dissolved in CH_2_Cl_2_ and added to acidic methanol (0.5 M HCl) while stirring. After standing, the supernatant was removed through centrifugation. The obtained white polymer was dried under vacuum at 50 °C until constant weight, and the yield was then calculated.

### 3.5. General Method for the Cycloaddition of CHO and CO_2_

A typical procedure for the cycloaddition reaction of CHO and CO_2_ was followed. An iron complex and cocatalyst were placed in a dry 50 mL stainless steel autoclave, and CHO (2 mL, 19.7 mmol) was added using a syringe under the protection of argon. The autoclave was sealed and immersed into an oil bath at the set temperature under stirring. Then, the CO_2_ was pressurized into the reactor to the specified pressure, and the reaction started. After stirring for the designated time, the reaction was stopped and cooled to ambient temperature. An aliquot for ^1^H NMR and FT-IR spectroscopy was taken to determine the conversion of CHO and product selectivity. The resulting mixture was transferred to a round-bottom flask. The unreacted CHO was removed under vacuum to obtain a pale-yellow liquid product.

### 3.6. UV Titration Procedure

The scanning range was set to 200~800 nm using a quartz cell with a 1 cm path length. The concentrations of ligand and Fe complex were 1 × 10^−4^ mol/L and 5 × 10^−5^ mol/L, respectively, with CH_2_Cl_2_ as the solvent. A solution of PPNCl or TBAB (2.5 × 10^−3^ mol/L) was prepared through a titration experiment. After each addition of 20 μL solution to 2 mL Fe complex solution, spectra were recorded.

## 4. Conclusions

A series of amino triphenolate iron complexes with various substituents were synthesized and fully characterized. Complexes C1–C4 exhibited efficient catalytic activity in the ring-opening polymerization of CHO through a bimetallic mechanism in the absence of co-catalysts. The activity of the four complexes under identical conditions followed the order of C1 ≈ C2 > C4 > C3, with the electron-withdrawing substituent having a positive impact on activity. The TOF was recorded as high as 12,623 h^−1^ at room temperature (25 °C) using C1 as the catalyst, and the number-average molecular weight of the polymerized product was determined to be 25.3 kg/mol. Furthermore, complexes C1–C4 demonstrated high selectivity in catalyzing the CHO/CO_2_ reaction to produce cis-cyclic carbonates in the presence of the co-catalyst TBAB. Mechanistic studies provided evidence of monometallic action in the presence of the co-catalyst.

## Figures and Tables

**Table 1:** Ring-opening polymerization of CHO catalyzed by complexes C1–C4.

Entry	Catalyst	CHO/Cat	Temp. (°C)	Time (min)	Yield (Conv. ^a^) (%)	TOF (h^−1^)	Mn (kg/mol)	Đ
1	C1	10,000/1	25	11	45.7 (46.9)	12,474	25.3	1.78
2	C2	10,000/1	25	12	48.5 (52.9)	12,134	26.4	1.74
3	C3	10,000/1	25	13	47.8 (51.8)	11,050	25.2	1.80
4	C4	10,000/1	25	15	56.3 (56.9)	11,262	23.4	1.88
5	C1	10,000/1	35	8	53.2	19,950	21.9	1.80
6	C2	10,000/1	35	11	48.0	13,101	20.8	1.95
7	C3	10,000/1	35	12	48.4	12,110	21.2	1.93
8	C4	10,000/1	35	12	50.9	12,701	22.5	1.89
9 ^b^	C4	10,000/1	25	60	0	0	-	-
10 ^c^	C4	10,000/1	25	60	0	0	-	-
11	C1	5000/1	25	3.5	53.1	22,773	23.4	1.83
12	C1	20,000/1	25	16	16.2	6062	22.9	2.02
13	C1	10,000/1	35	8	56.2	21,058	19.1	2.08
14	C1	20,000/1	35	17	48.6	17,165	18.7	2.10
15	C1	40,000/1	35	60	28.9	5780	17.6	2.18
16	C1	10,000/1	45	3	38.0	38,000	19.1	2.08
17	C1	10,000/1	55	2.5	36.0	42,911	17.8	2.26

^a^ Determined by ^1^H NMR spectroscopy. ^b^ Reaction was briefly exposed to air at initiation. ^c^ Cat/PPNCl molar ratio is 1/2.

**Table 2:** Effect of solvent type and volume on polymerization ^a^.

Entry	Solvent	Volume (mL)	Time (min)	Yield (%)	TOF (h^−1^)	Mn (kg/mol)	Đ
1	THF	0.5	45	-	-	-	-
2	Hexane	0.5	45	48.8	3251	20.2	1.98
3	Toluene	0.5	30	49.4	4937	21.7	1.93
4	Toluene	1	45	33.9	2257	20.8	1.96
5	Dichloromethane	0.5	30	55.5	5545	25.5	1.82
6	Dichloromethane	1	45	61.1	4072	24.3	1.85
7	Dichloromethane	1	90	69.0	2300	21.5	1.96
8	Dichloromethane	1	135	70.8	1573	20.9	2.10
9	1,2-Dichloroethane	0.5	30	58.9	5892	24.8	1.85

^a^ Reaction conditions: CHO/C1 = 10,000/1, 25 °C.

**Table 3:** Effect of cocatalyst types and proportions on catalytic performance ^a^.

Entry	Cat.	Cocat	CHO/Cat/Cocat	Conv. (%) ^b^	PCHC/*cis*-CHC ^c^	PCHO (%)
1	C4	-	2000/1/0	20	-	99
2	-	TBAB	2000/0/8	17	1/99	-
3	C4	PPNCl	2000/1/8	67	1/99	<1
5	C4	TBAB	2000/1/2	46	2/98	2
6	C4	TBAB	2000/1/4	74	1/99	<1
7	C4	TBAB	2000/1/8	80	1/99	<1
8	C1	TBAB	2000/1/8	71	1/99	<1
9	C2	TBAB	2000/1/8	73	1/99	<1
10	C3	TBAB	2000/1/8	74	1/99	<1

^a^ Reaction conditions: 16 h, 3 MPa CO_2_, 100 °C. ^b^ Calculated based on the integral of the NMR results. ^c^ Calculated from NMR and IR results.

**Table 4:** Effect of reaction conditions on the cycloaddition of CHO with CO_2_ ^a^.

Entry	T (°C)	Time (h)	P (MPa)	Conv. (%)	PCHC/cis-CHC (%)	PCHO (%)
1	100	4	3	37	1/99	<1
2	100	8	3	50	1/99	<1
3	100	12	3	65	1/99	<1
4	100	16	3	80	1/99	<1
5 ^a^	100	12	3	48	1/99	<1
6 ^b^	100	12	3	77	1/99	<1
7	80	12	3	47	2/98	<1
8	120	12	3	85	1/99	<1
9	100	12	0.1	30	1/99	3
10	100	12	1.5	40	1/99	<1
11	100	12	4.5	68	1/99	<1

^a^ Reaction conditions: CHO/Cat/TBAB = 2000/1/8, Cat: C4. ^b^ CHO/Cat/TBAB = 4000/1/8. ^c^ CHO/Cat/TBAB = 1000/1/8.

## Data Availability

Data are contained within the article and Supplementary Materials.

## Supplementary Materials

The following supporting information can be downloaded at: https://www.mdpi.com/article/10.3390/molecules29092139/s1, Scheme S1: Synthesis of 2,4-dichloro-6-chloromethylphenol. Figure S1: ^1^H NMR spectrum of ligand-1; Figure S2: ^13^C NMR spectrum of ligand-1; Figure S3: ^1^H NMR spectrum of ligand-1; Figure S4: ^13^C NMR spectrum of ligand-2; Figure S5: ^1^H NMR spectrum of ligand-3; Figure S6: ^13^C NMR spectrum of ligand-3; Figure S7: ^1^H NMR spectrum of ligand-4; Figure S8: ^13^C NMR spectrum of ligand-4; Figure S9: HRMS of complex-**1** (positive mode); Figure S10: HRMS of complex-**2** (positive mode); Figure S11: HRMS of complex-**3** (positive mode); Figure S12: HRMS of complex-**4** (positive mode); Figure S13: UV-vis spectra of ligands 1–4 and complexes **1**–**4**; Figure S14: IR spectra of ligands 1–4 and complexes **1**–**4**; Figure S15: UV-vis spectra of complexes **1**–**4** titrated with 500 equivalent of CHO; Figure S16: HRMS of (a) complex-**1** and (b) complex-**4** and PPNCl in a molar ratio of 1:2; Figure S17: IR spectrum of PCHO; Figure S18: 1H NMR spectrum of PCHO; Figure S19: DSC polts of PCHO; Figure S20: TG and DTG curves of PCHO; Figure S21: UV spectrum of complex C4 with different equivalent TBAB; Figure S22: HRMS spectrum of complex C4 and TBAB in a molar ratio of 1:2. Figure S23: ^1^H NMR spectrum of the crude reaction mixture of CHO/CO_2_ copolymerization in CDCl_3_. Figure S24: FT-IR spectrum of crude reaction mixture of CHO/CO_2_.
