# Supplementary material for: Ring-Opening Polymerization of Cyclohexene Oxide and Cycloaddition with CO2 Catalyzed by Amine Triphenolate Iron(III) Complexes
Source: Molecules. 2024 May 4;29(9):2139. doi: 10.3390/molecules29092139 (PMC11085797; doi:10.3390/molecules29092139)
Supplement: Supplementary file 1 [file molecules-29-02139-s001.zip › molecules-2992707-supplementary.pdf]

Supporting information

Ring opening polymerization of cyclohexene oxide and cycloaddition with CO_2 catalyzed by amine triphenol iron(III) complexes

Peng Li[a], Sixuan Li[a], Xin Dai[a], Shifeng Gao[b], Zhaozheng Song[a]*, and Qingzhe Jiang[a,c]*

[a]State Key Laboratory of Heavy Oil Processing, College of Science, China University of Petroleum, Beijing 102249, PR China

[b]CNPC Engineering Technology R&D Company Ltd., Beijing 102206, China

[c]School of International Trade and Economics, University of International Business and Economics, Beijing 100029, PR China

CONTENTS

Supporting information 1

CONTENTS 2

Experimental section 4

Scheme S1. Synthesis of 2,4-dichloro-6-chloromethylphenol 4

Figure S1. ^{1}H NMR spectrum of ligand-1 5

Figure S2. ^{13}C NMR spectrum of ligand-1 5

Figure S3. ^{1}H NMR spectrum of ligand-2 6

Figure S4. ^{13}C NMR spectrum of ligand-2 6

Figure S5. ^{1}H NMR spectrum of ligand-3 7

Figure S6. ^{13}C NMR spectrum of ligand-3 7

Figure S7. ^{1}H NMR spectrum of ligand-4 8

Figure S8. ^{13}C NMR spectrum of ligand-4 8

Figure S9. HRMS of complex-1 (positive mode) 9

Figure S10. HRMS of complex-2 (positive mode) 9

Figure S11. HRMS of complex-3 (positive mode) 10

Figure S12. HRMS of complex-4 (positive mode) 10

Figure S13. UV-vis spectra of ligands 1-4 and complexes 1-4 11

Figure S14. IR spectra of ligands 1-4 and complexes 1-4 11

Figure S15. UV-vis spectra of complexes 1-4 titrated with 500 equivalent of CHO 12

Figure S16. HRMS of (a) complex-1 and (b) complex-4 and PPNCl in a molar ratio of 1:2 (negative mode) 13

Figure S17. IR spectrum of PCHO 14

Figure S18. ^{1}H NMR spectrum of PCHO 15

Figure S19. DSC polts of PCHO (Table 1) 16

Figure S20 TG and DTG curves of PCHO 16

Figure S21. UV spectrum of complex C4 with different equivalent TBAB 17

Figure S22. HRMS spectrum of complex C4 and TBAB in a molar ratio of 1:2 (Negative mode) 17

Analysis of CHO ring opening polymerization 18

Analysis of CHO/CO_2 cycloaddition reactions: 18

Reference 19

Experimental section

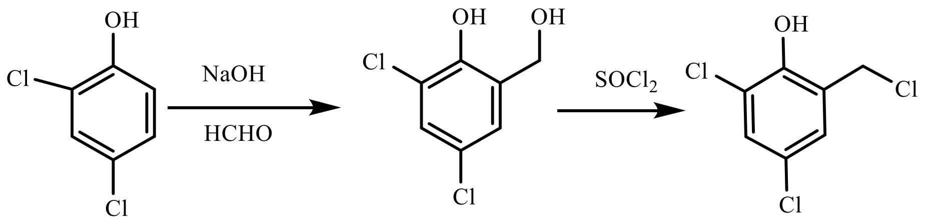

Scheme S1. Synthesis of 2,4-dichloro-6-chloromethylphenol

Synthesis of 2, 4-dichloro-6-hydroxymethylphenol:

15 mL of deionized water was added to the flask, followed by the addition of sodium hydroxide (1.3 g, 63 mmol). The mixture was stirred for 15 min until the sodium hydroxide had completely dissolved. Next, 2,4-dichlorophenol (5 g, 100 mmol) was added and dissolved in the reaction mixture. Formaldehyde solution (37%-40%, 6.0 mL, 167.6 mmol) was then added, and the mixture was stirred at 50 ℃ for 48 hours. During this process, the color of the reaction mixture gradually changed from light yellow to yellow to dark red. After the reaction stopped, the resulting solution was transferred to a beaker, and its pH was adjusted to about 3 with hydrochloric acid while stirring to give a deep red oily substance. This oily substance was extracted with dichloromethane (CH_2Cl_2) three times using 50 mL portions. The CH_2Cl_2 extract was washed with saturated salt water and dried with anhydrous sodium sulfate. Finally, the CH_2Cl_2 extract was recrystallized to give a white solid product (8.3 g, 76.7% yield). ^{1}H NMR (500 MHz, $CDCl_3$) δ 7.28 (s, 1H, Ar*H*), 7.11 (s, 1H, Ar*H*), 6.70 (s, 1H, Ar*H*), 4.76 (s, 2H, ArCH_2OH), 2.44 (s, 1H, ArCH_2*OH*).

Synthesis of 2,4-dichloro-6-chloromethylphenol:

Under an argon atmosphere, a 100 mL reaction flask was charged with 30 mL of anhydrous dichloromethane, 2,4-di-tert-butyl-6-hydroxymethylphenol (0.92 g, 5 mmol) was added and stirred until completely dissolved. Subsequently, sulfoxide chloride (0.5 mL, 6.67 mmol) was slowly added dropwise and the reaction mixture was kept at 30 ℃ for 10 hours. The solvent and unreacted sulfoxide chloride were removed using a rotary evaporator, and the white solid (0.98 g, 98%) was obtained by vacuum drying overnight [1]. ^{1}H NMR (500 MHz, $CDCl_3$) δ 7.32 (d, *J* = 2.4 Hz, 1H, Ar*H*), 7.28 (d, *J* = 2.3 Hz, 1H, Ar*H*), 5.77 (s, 1H, Ar*OH*), 4.62 (s, 2H, ArCH_2Cl).

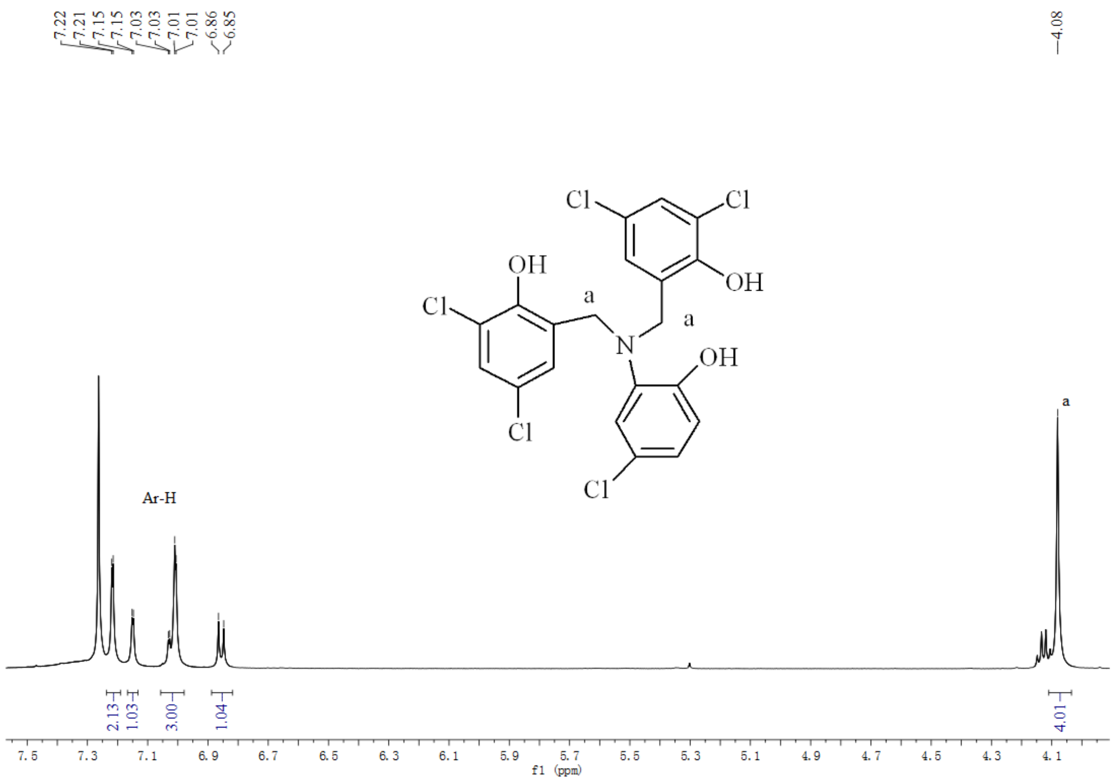

Figure S1. ^{1}H NMR spectrum of ligand-1

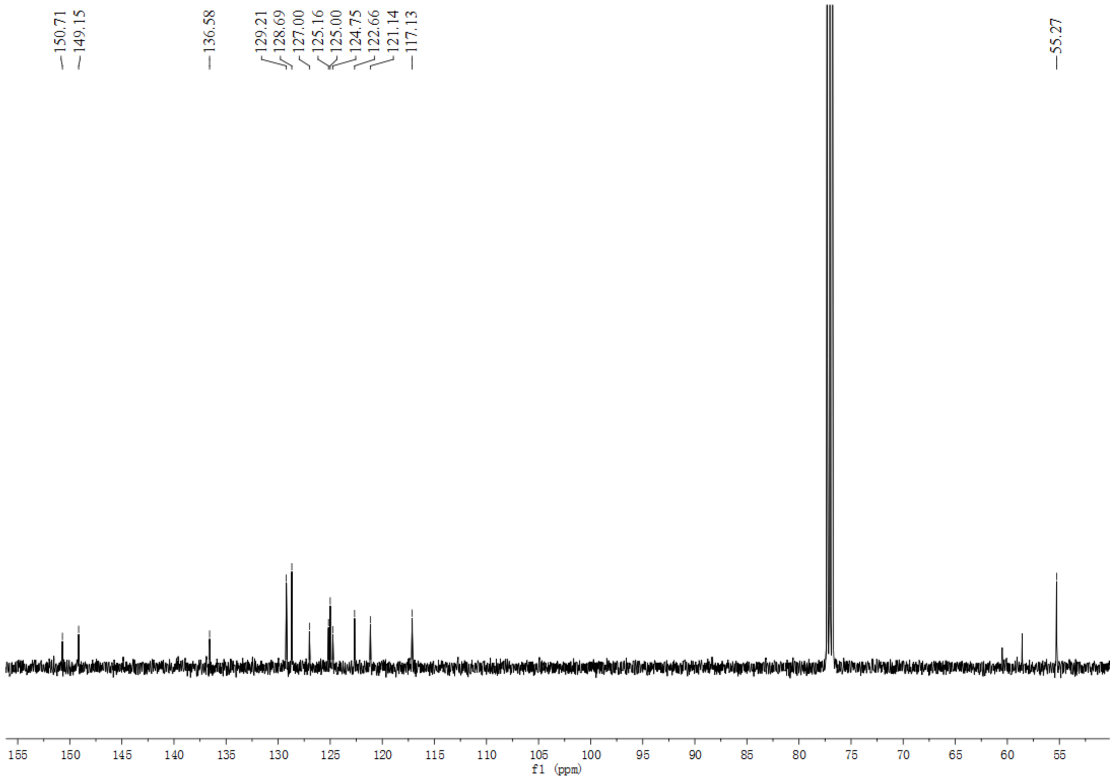

Figure S2. ^{13}C NMR spectrum of ligand-1

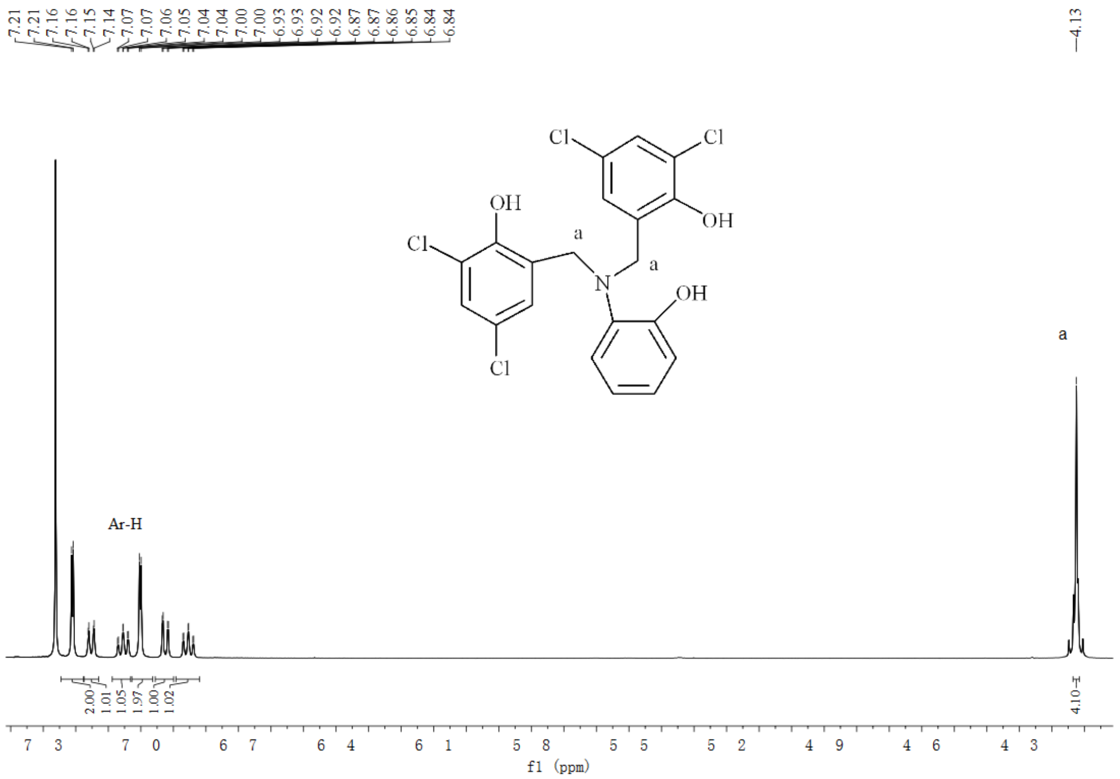

Figure S3. ^{1}H NMR spectrum of ligand-2

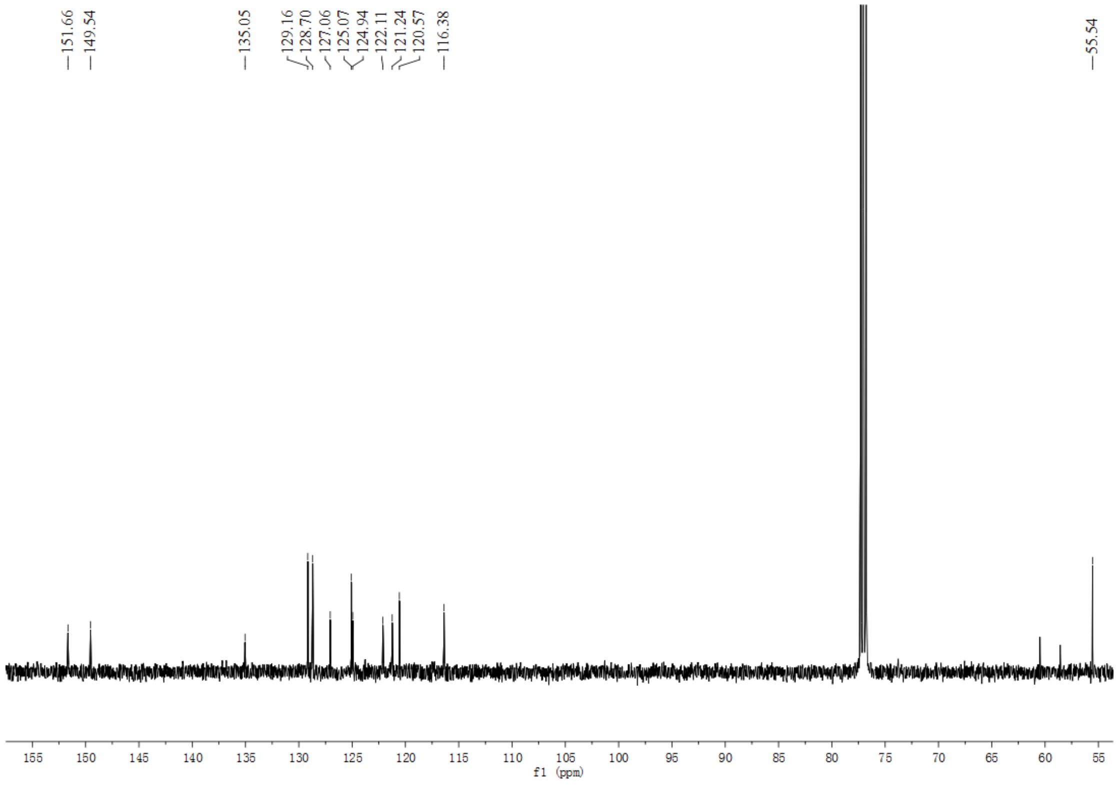

Figure S4. ^{13}C NMR spectrum of ligand-2

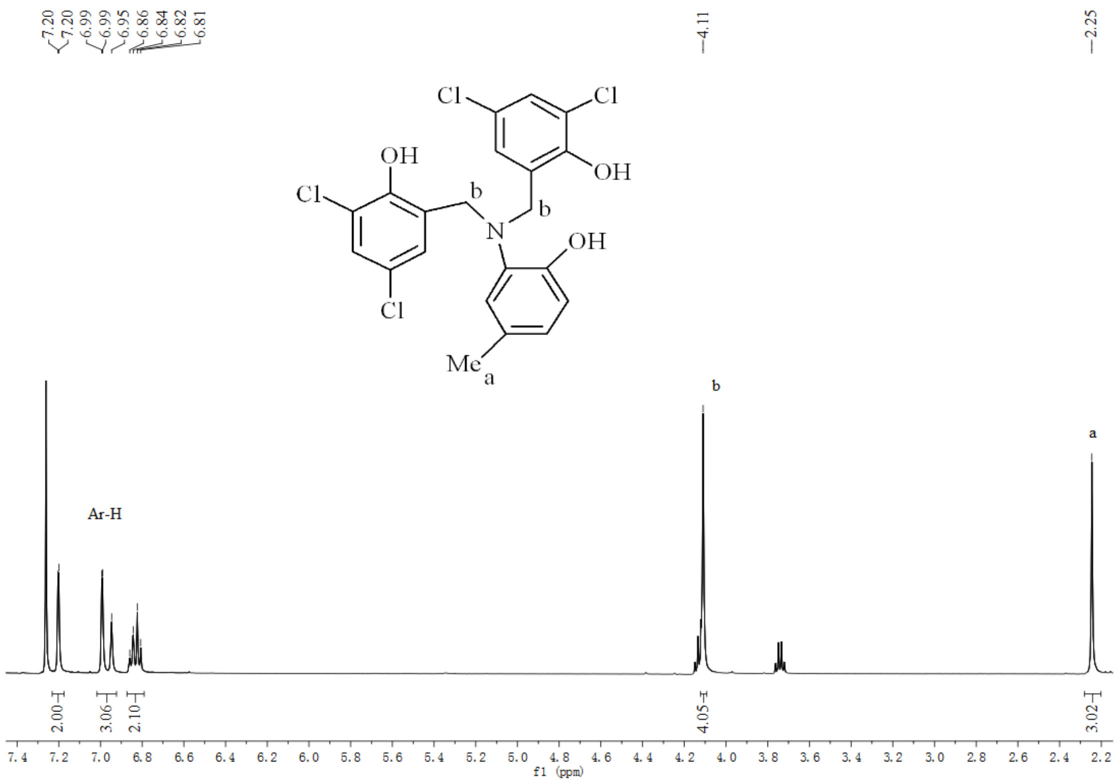

Figure S5. ^{1}H NMR spectrum of ligand-3

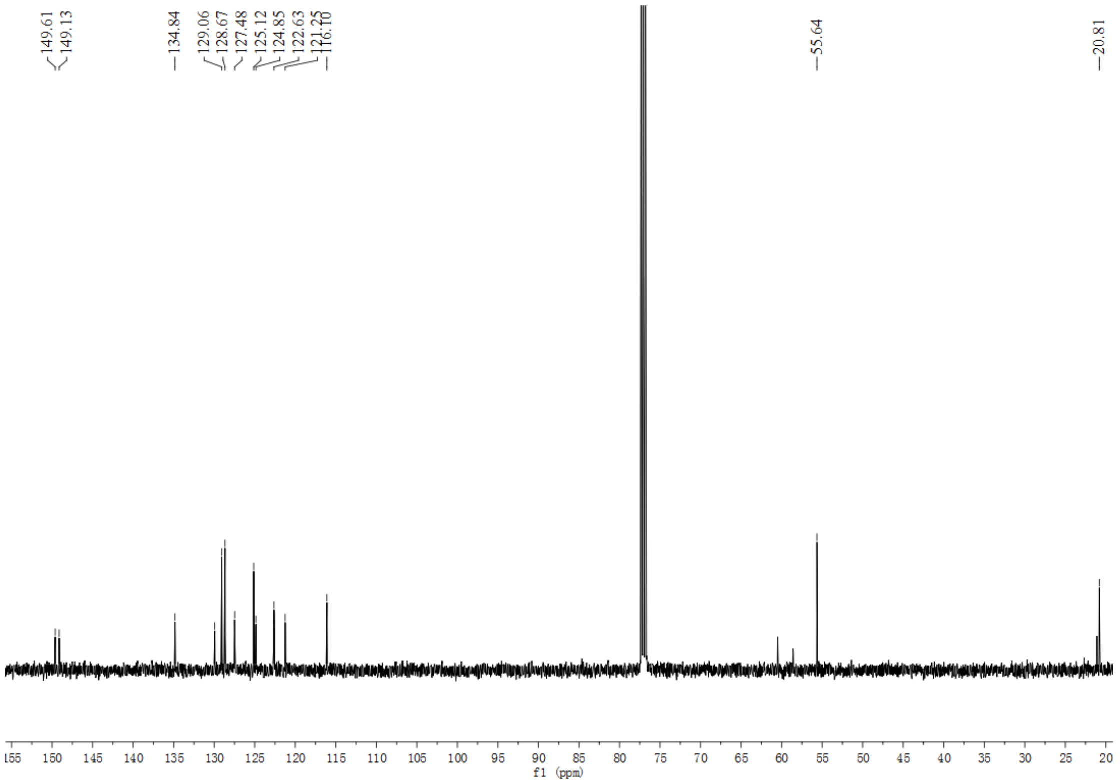

Figure S6. ^{13}C NMR spectrum of ligand-3

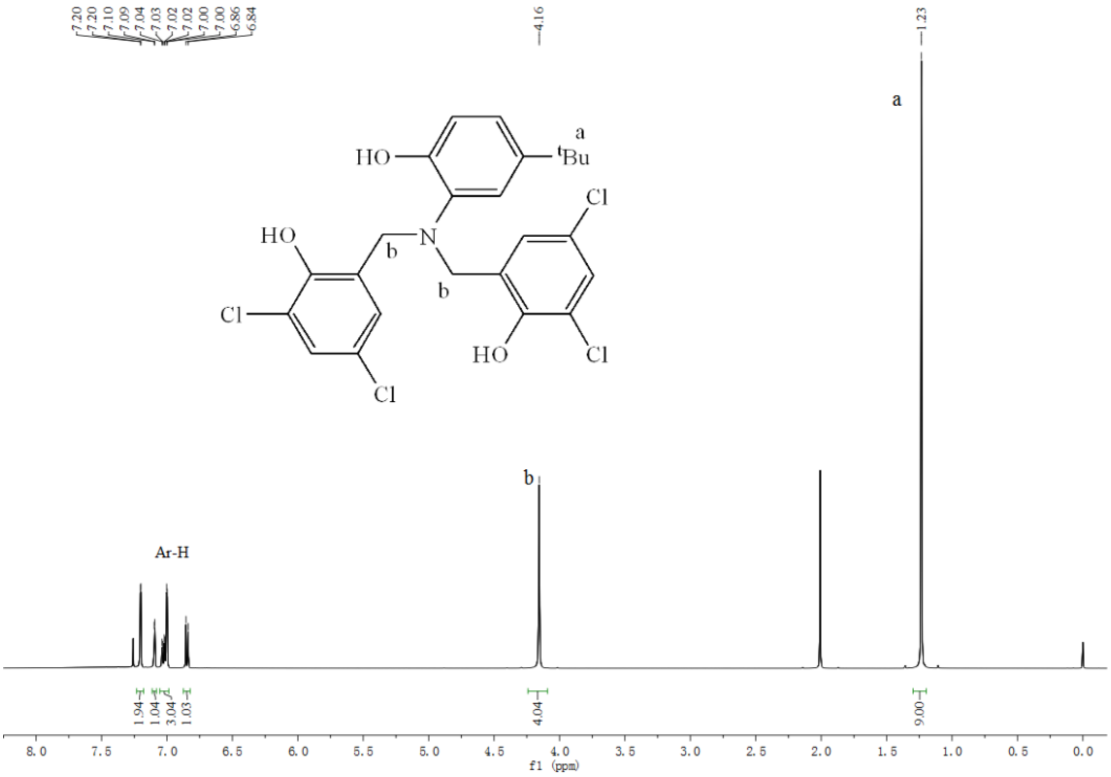

Figure S7. ^{1}H NMR spectrum of ligand-4

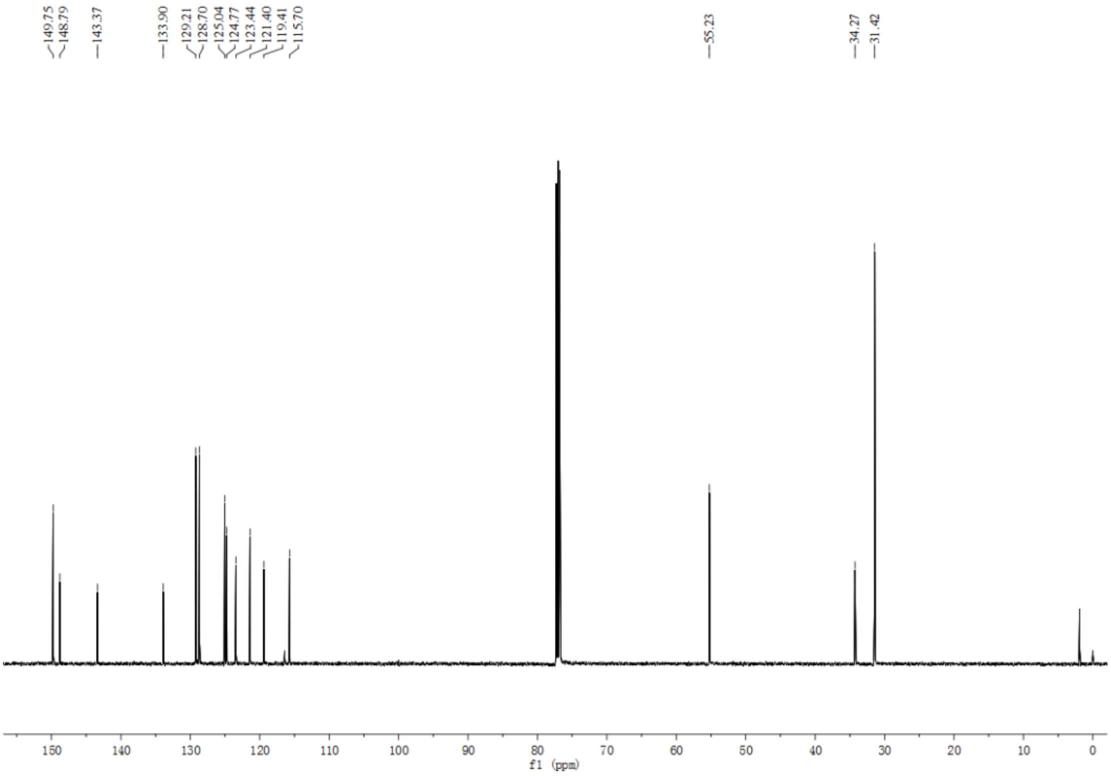

Figure S8. ^{13}C NMR spectrum of ligand-4

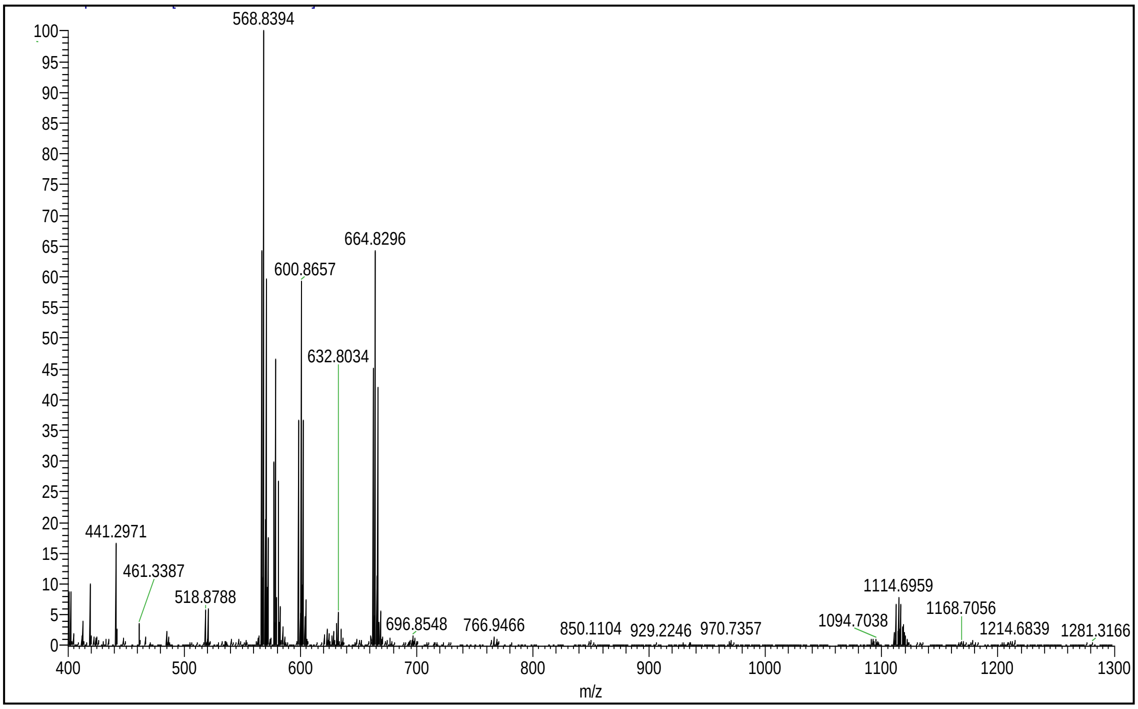

Figure S9. HRMS of complex-1 (positive mode)

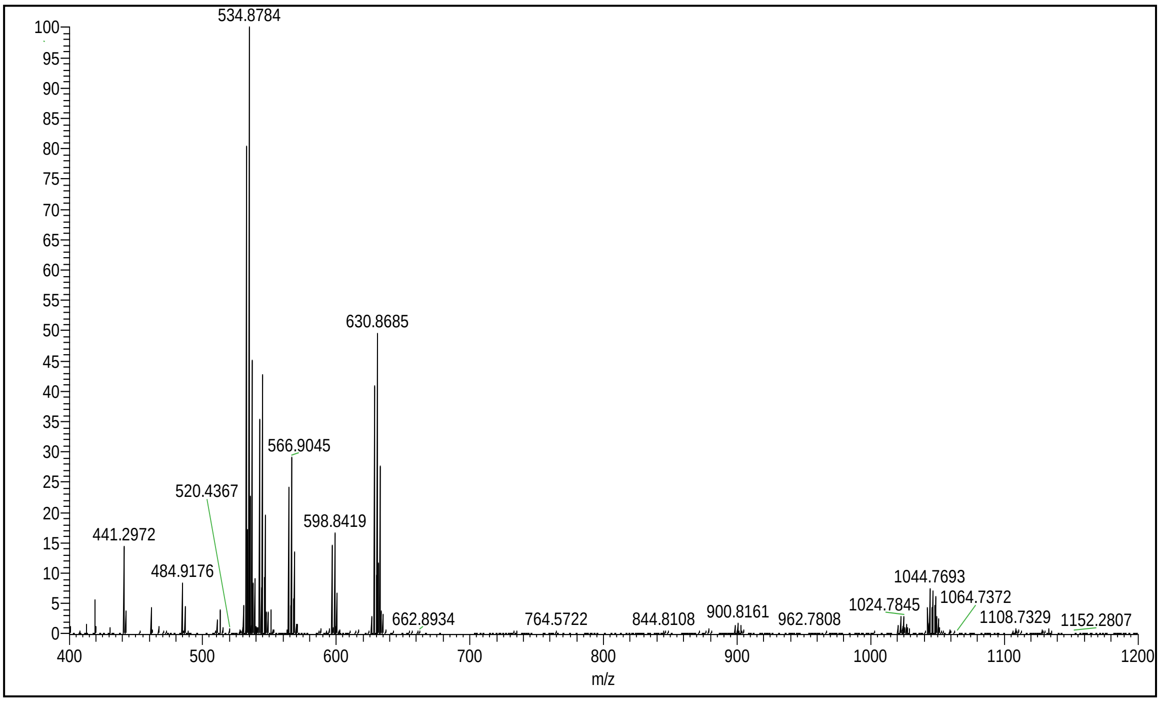

Figure S10. HRMS of complex-2 (positive mode)

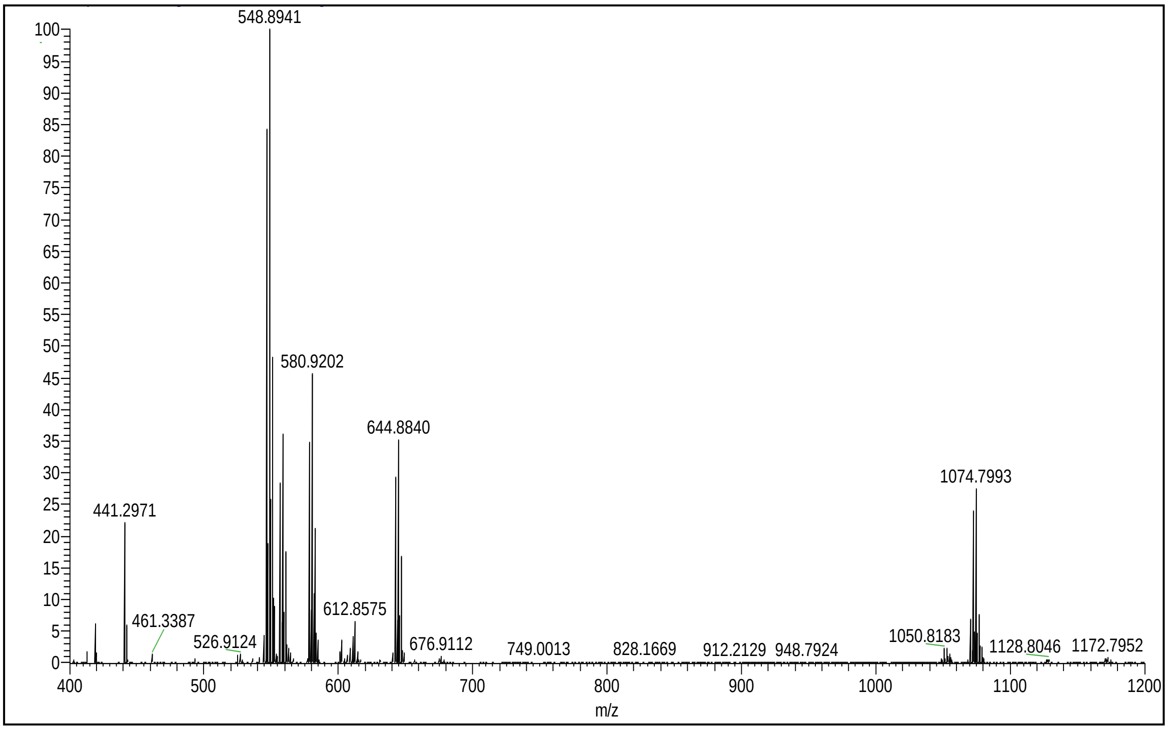

Figure S11. HRMS of complex-3 (positive mode)

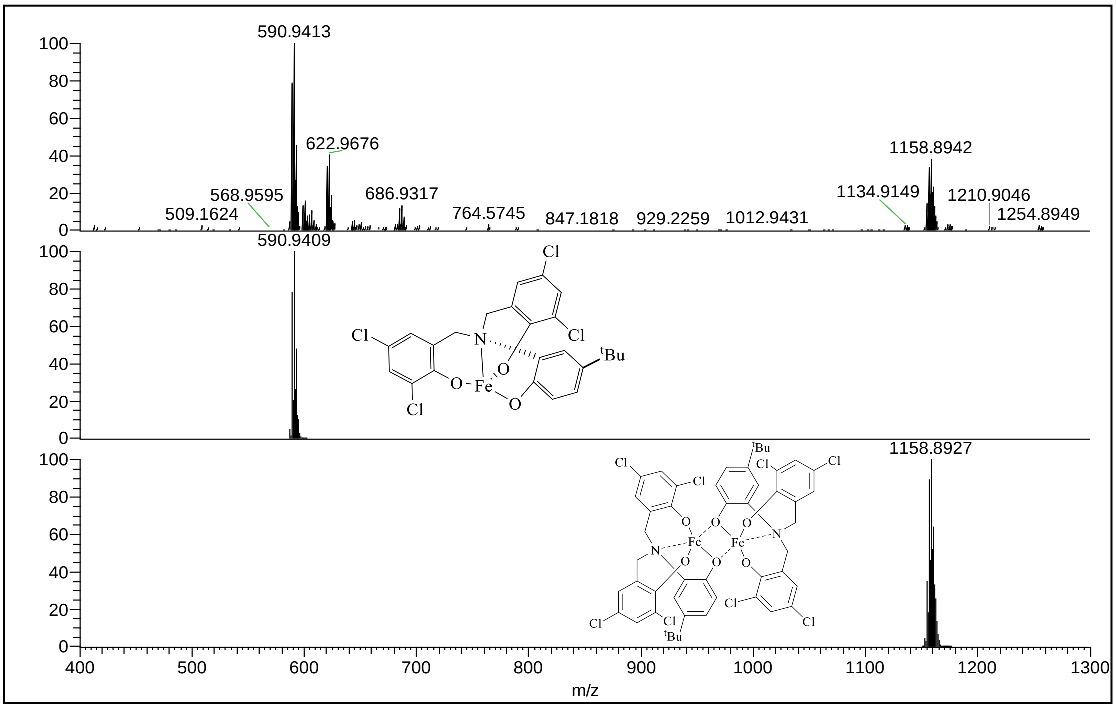

Figure S12. HRMS of complex-4 (positive mode)

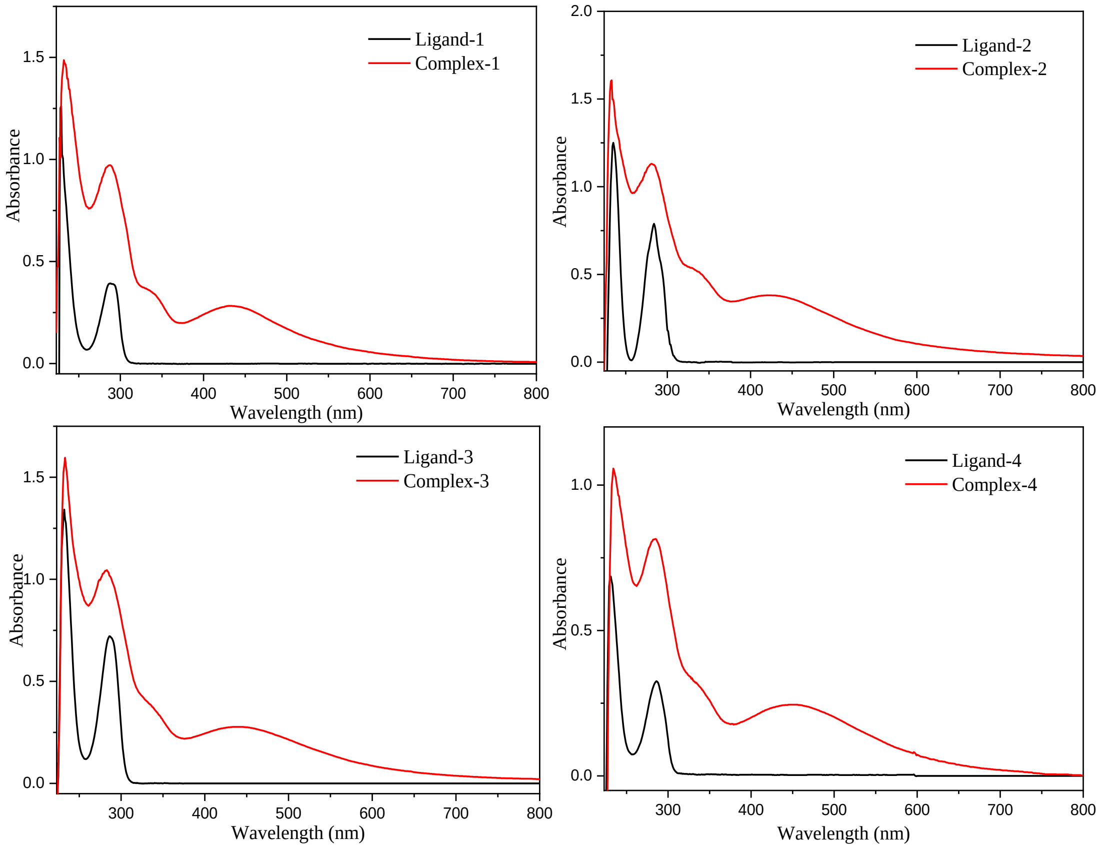

Figure S13. UV-vis spectra of ligands 1-4 and complexes 1-4

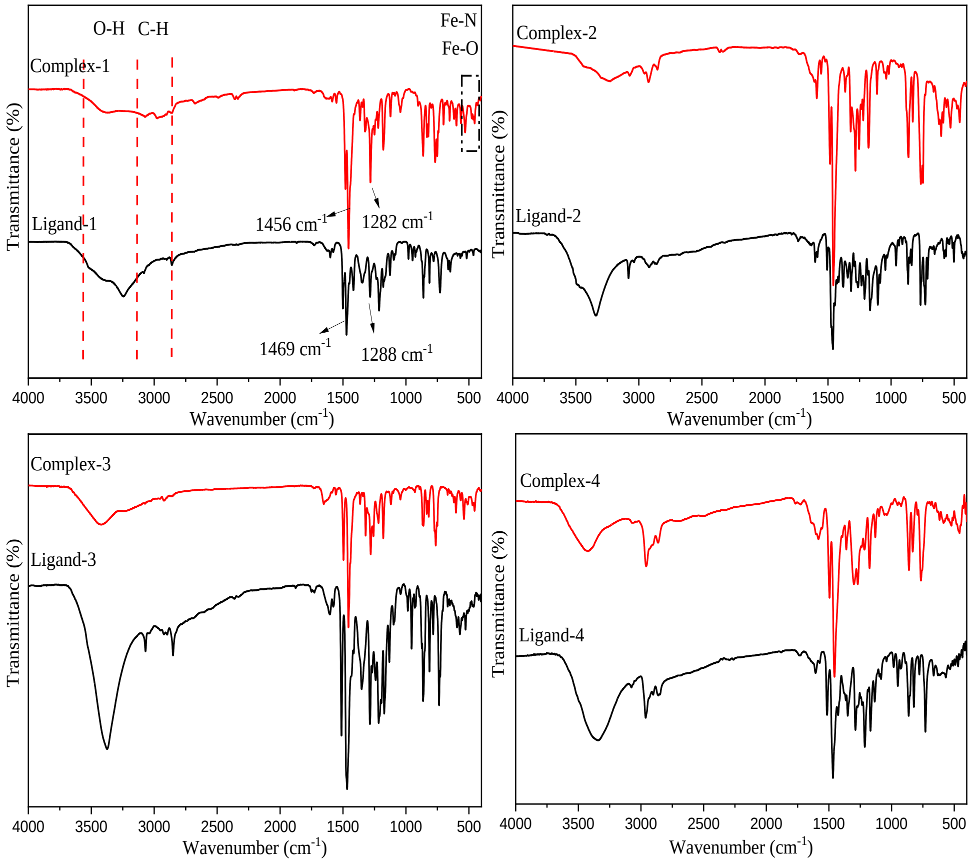

Figure S14. IR spectra of ligands 1-4 and complexes 1-4

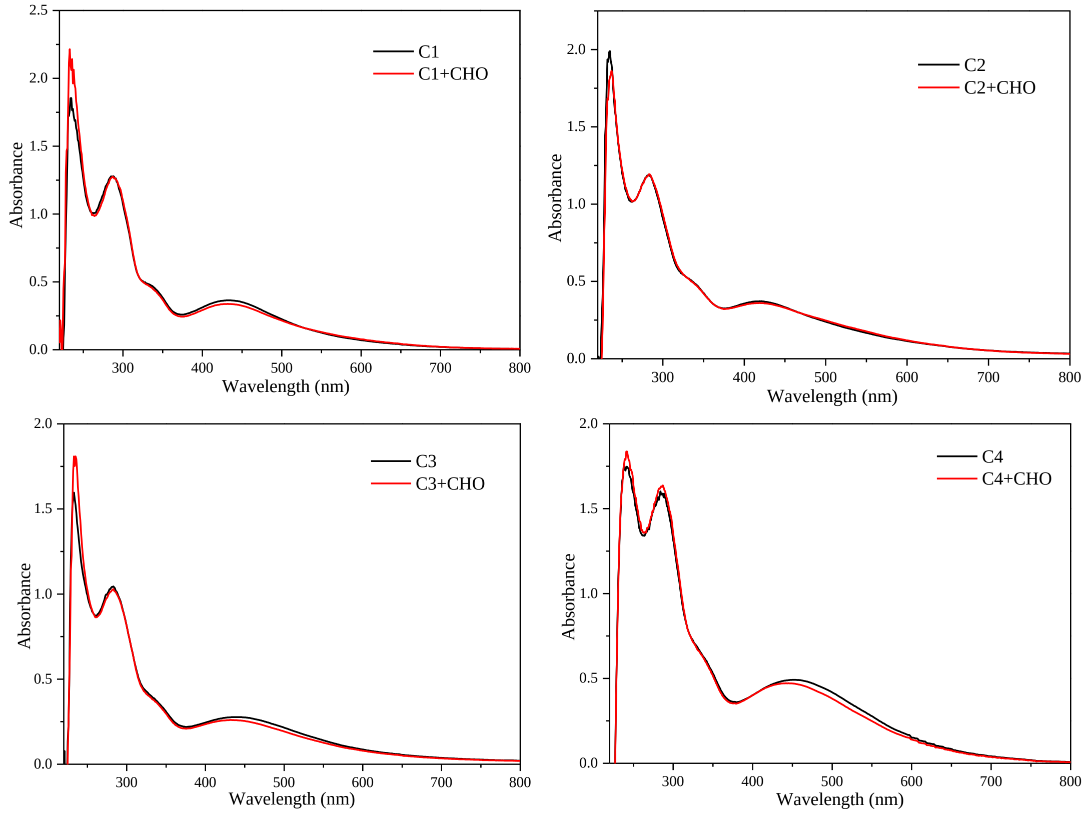

Figure S15. UV-vis spectra of complexes 1-4 titrated with 500 equivalent of CHO

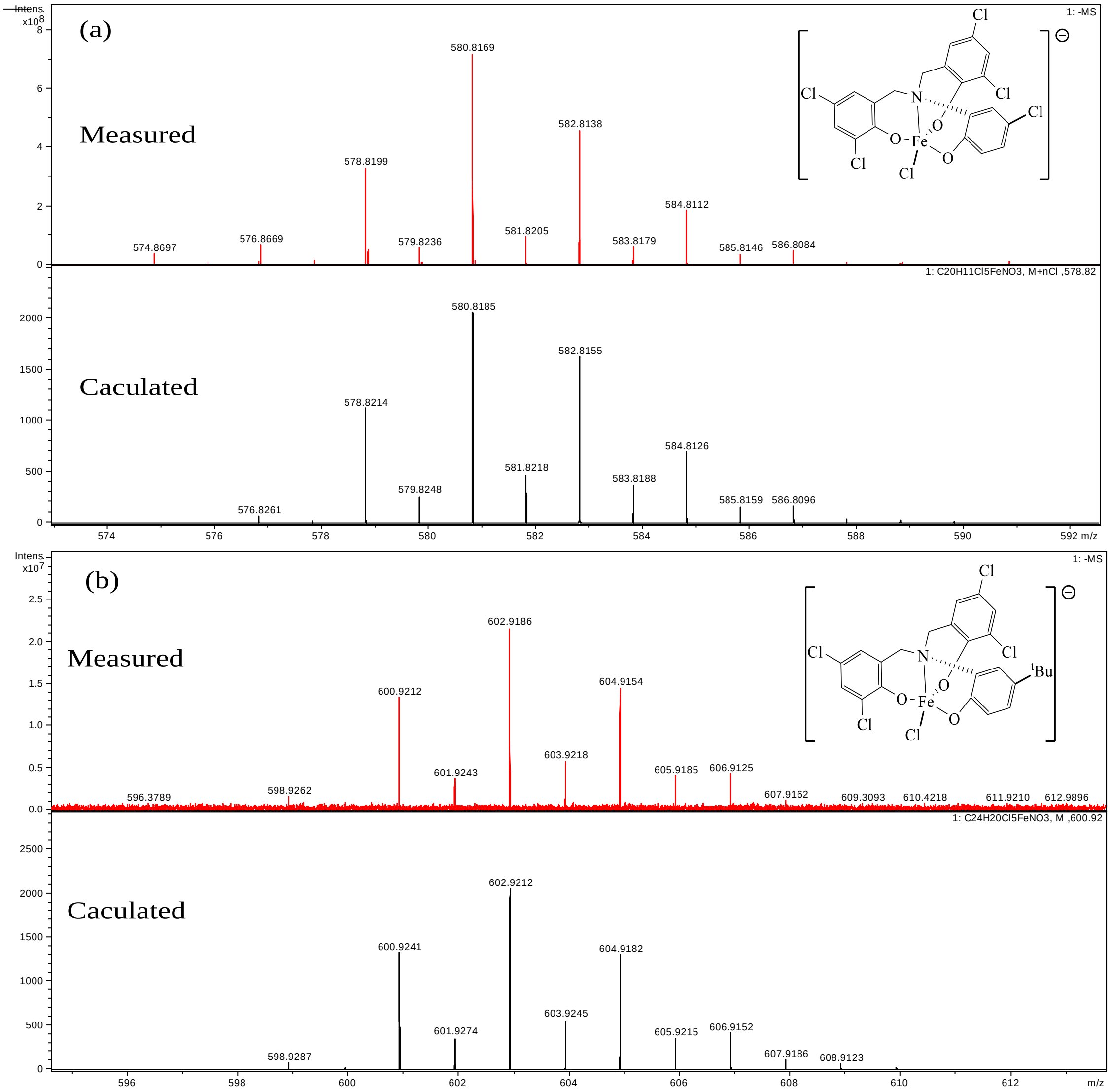

Figure S16. HRMS of (a) complex-1 and (b) complex-4 and PPNCl in a molar ratio of 1:2 (negative mode)

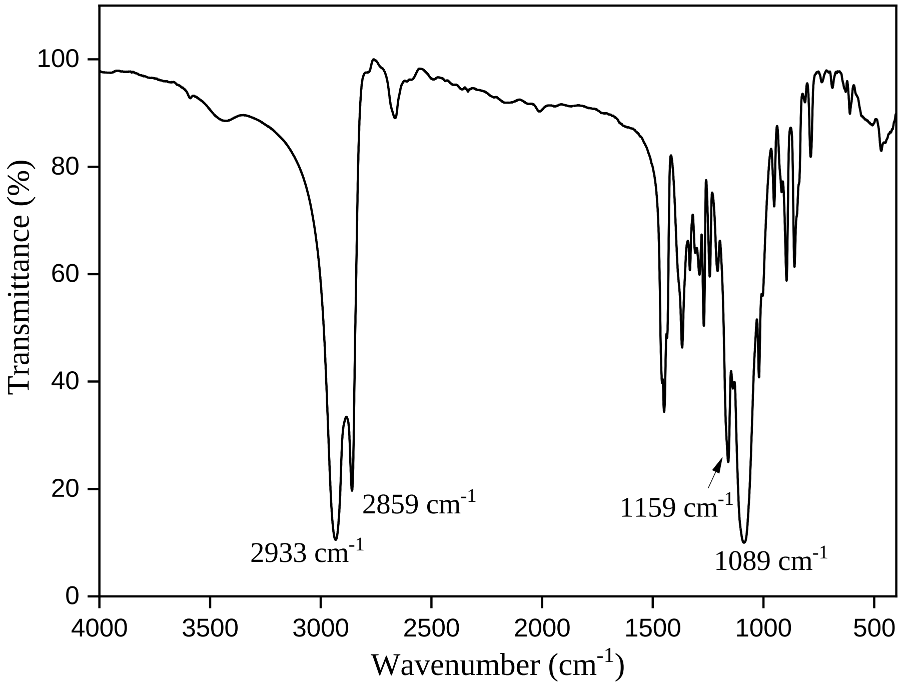

Figure S17. IR spectrum of PCHO

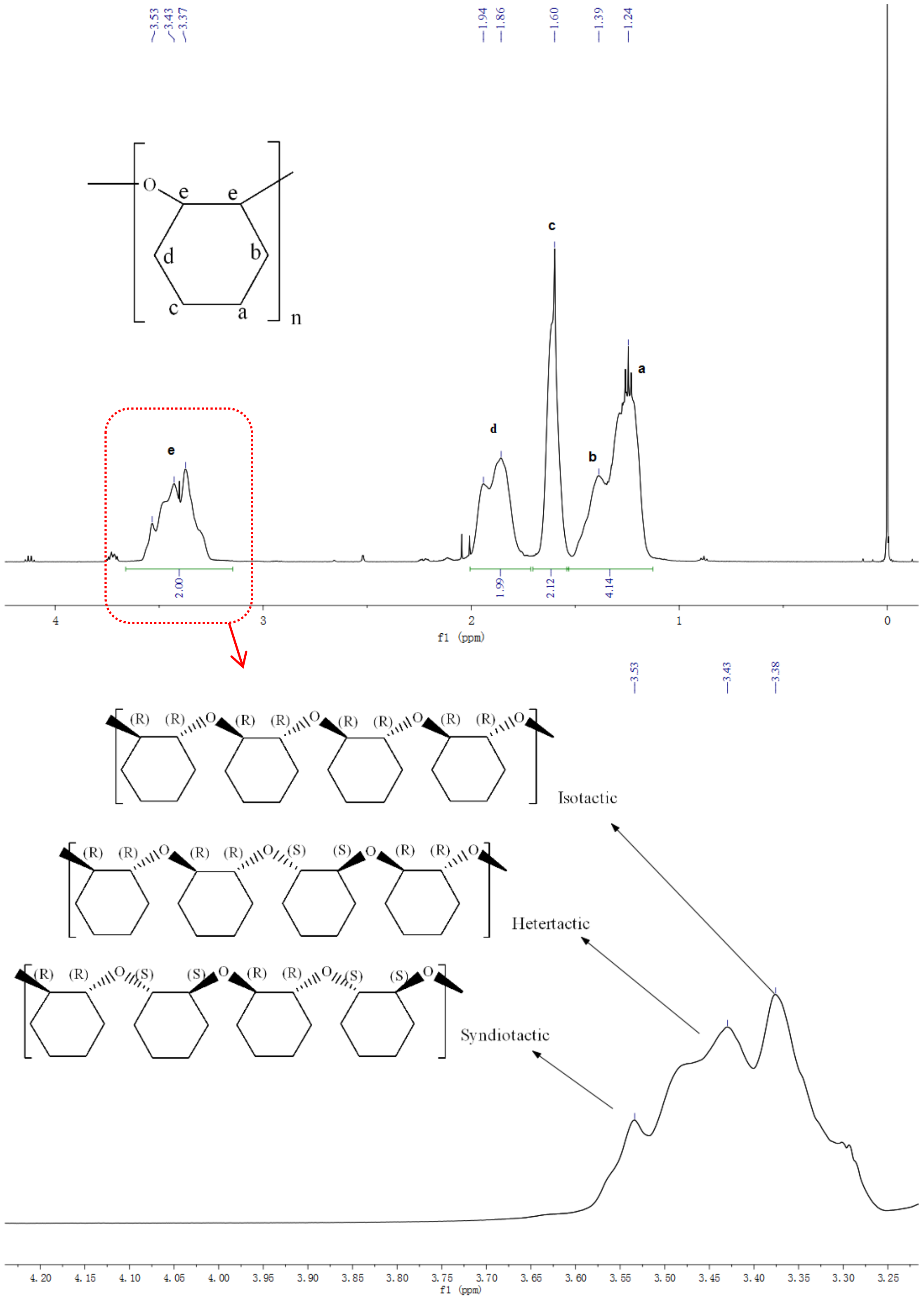

Figure S18. ^{1}H NMR spectrum of PCHO

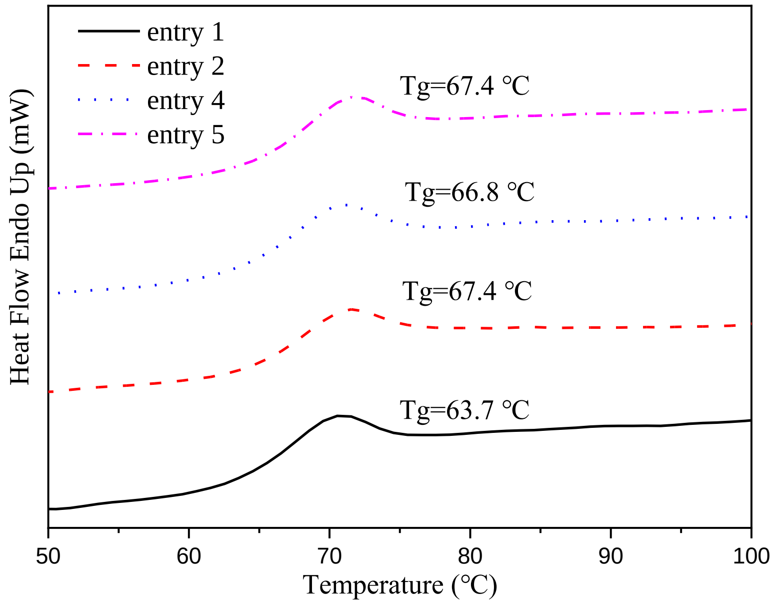

Figure S19. DSC polts of PCHO (Table 1)

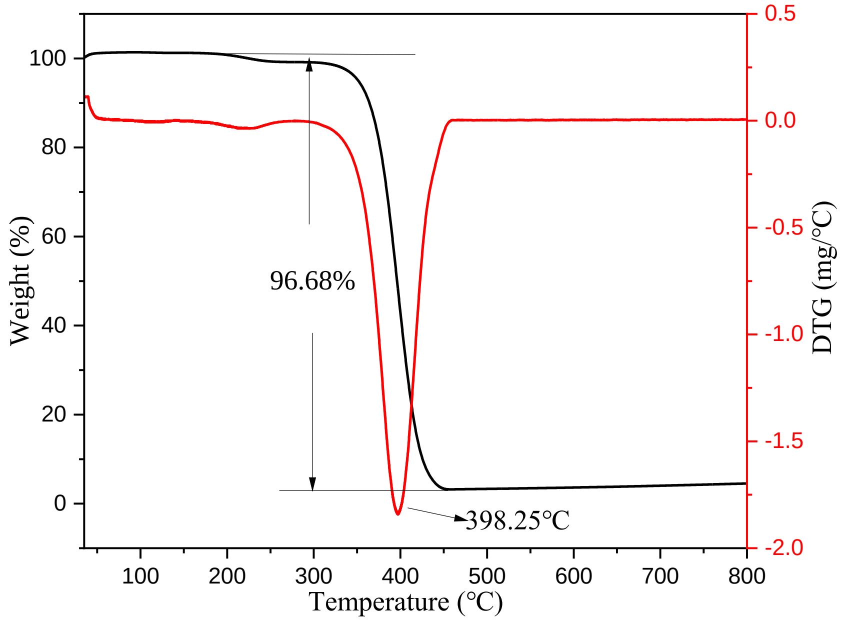

Figure S20 TG and DTG curves of PCHO

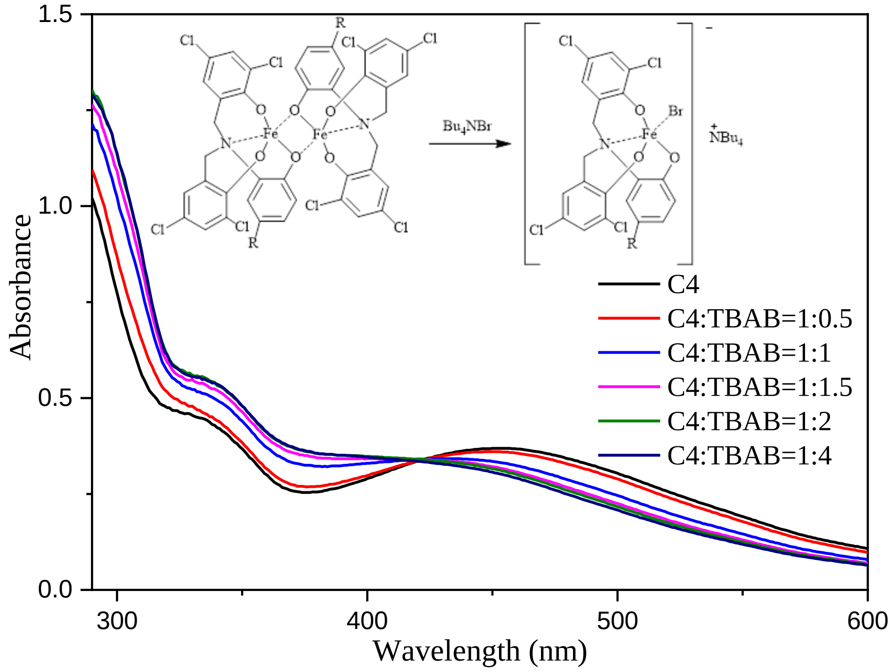

Figure S21. UV spectrum of complex C4 with different equivalent TBAB

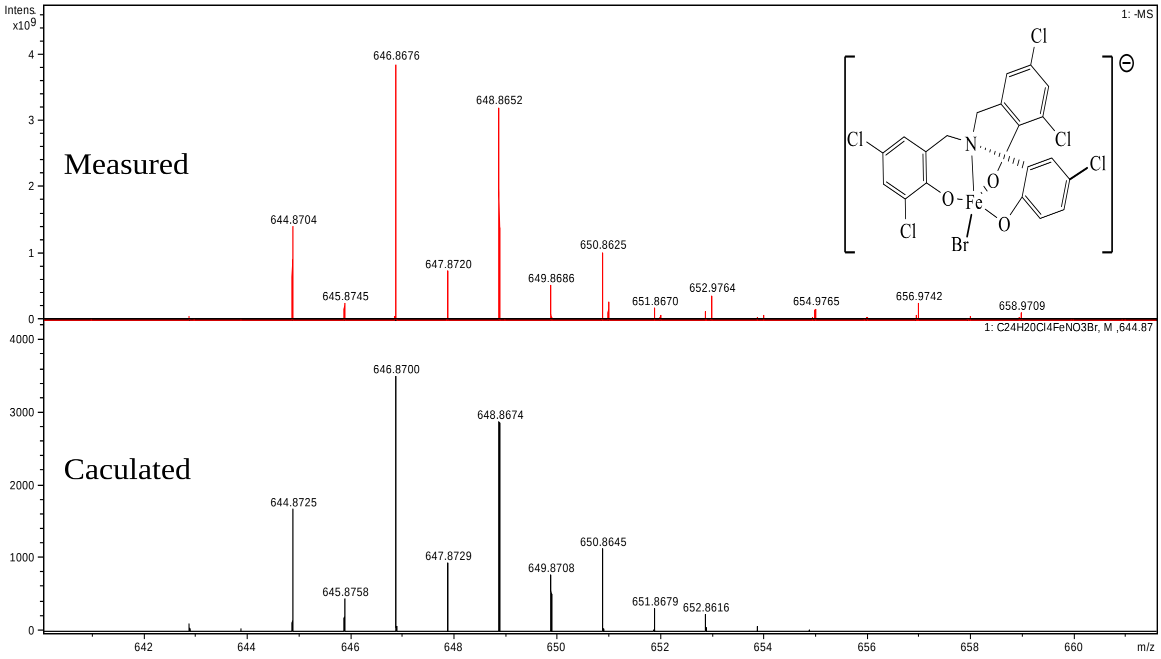

Figure S22. HRMS spectrum of complex C4 and TBAB in a molar ratio of 1:2 (Negative mode)

Analysis of CHO ring opening polymerization

$$\text{Conversion of CHO (\%)} = \frac{I_{3.40}}{I_{3.12}+I_{3.40}} \times 100$$

$$\text{Yield (\%)} = \frac{\text{Weight of CHO}}{\text{Weight of product}} \times 100$$

$$\text{TOF (h}^{-1}\text{)} = \frac{\text{mol of CHO consumed}}{\text{mol of Fe center} \cdot \text{h}}$$

Analysis of CHO/CO_2 cycloaddition reactions:

Examples of ^{1}H NMR spectra used for determining the conversion of CHO.

$$\text{Conversion of CHO (\%)} = \frac{I_{4.55\text{-}4.75}+I_{4.63}+I_{4.09}+I_{3.40}}{I_{4.55\text{-}4.75}+I_{4.63}+I_{4.09}+I_{3.40}+I_{3.12}} \times 100\%$$

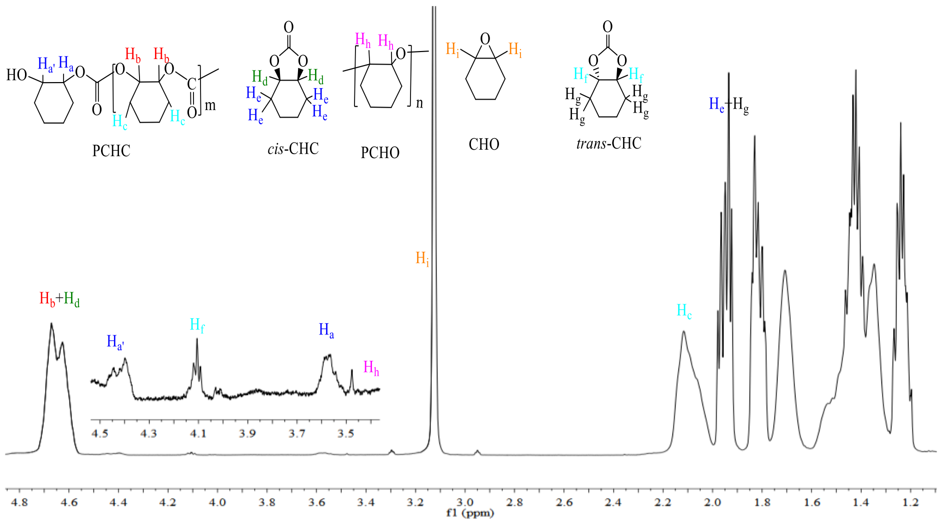

Figure S23. ^{1}H NMR spectrum of the crude reaction mixture of CHO/CO_2 copolymerization in $CDCl_3$

Examples of FT-IR spectra used for determining the ratio between cyclic cyclohexene carbonate (CHC) and poly(cyclohexene carbonate) (PCHC):

$$A_{1742} = \log\frac{T_0}{T_1}$$

$$A_{1802} = \log\frac{T_0}{T_2}$$

$$cis\text{-CHC } (\%) = \frac{A_{1742}}{A_{1742}+A_{1802}} \times 100$$

where T_0 is transmittance of the baseline and T_1 is transmittance of the C=O band at 1742 cm^{-1}, and T_2 is transmittance of the C=O band at 1802 cm^{-1}

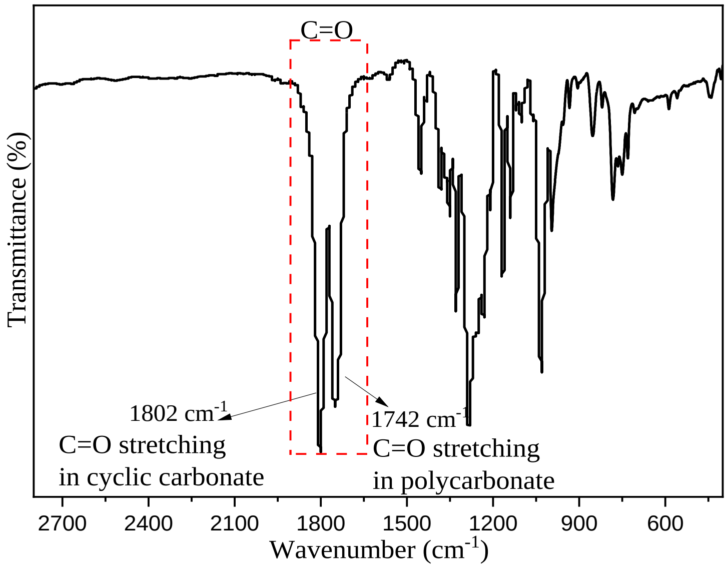

Figure S24. FT-IR spectrum of crude reaction mixture of CHO/CO_2

Reference

[1] Basu D, Allard M M, Xavier F R, et al. Modulation of electronic and redox properties in phenolate-rich cobalt(iii) complexes and their implications for catalytic proton reduction[J]. Dalton Transactions, 2015,44(7):3454-3466.
